# Cross-Platform Evaluation of Commercially Targeted and Untargeted Metabolomics Approaches to Optimize the Investigation of Psychiatric Disease

**DOI:** 10.3390/metabo11090609

**Published:** 2021-09-08

**Authors:** Lauren E. Chaby, Heather C. Lasseter, Kévin Contrepois, Reza M. Salek, Christoph W. Turck, Andrew Thompson, Timothy Vaughan, Magali Haas, Andreas Jeromin

**Affiliations:** 1 Cohen Veterans Bioscience, New York, NY 10018, USA; lauren.chaby@cohenbio.org (L.E.C.); lasseterh@gmail.com (H.C.L.); andrew.thompson@cohenbio.org (A.T.); timothy.vaughan@cohenbio.org (T.V.); magali.haas@cohenbio.org (M.H.); 2 Department of Genetics, Stanford University School of Medicine, Stanford, CA 94305, USA; kcontrep@stanford.edu; 3 International Agency for Research on Cancer, Nutrition and Metabolism Branch, World Health Organisation, 150 Cours Albert Thomas, CEDEX 08, 69372 Lyon, France; r7salek@gmail.com; 4 Max Planck Institute of Psychiatry, Proteomics and Biomarkers, 80804 Munich, Germany; turck@psych.mpg.de

**Keywords:** posttraumatic stress disorder (PTSD), metabolomics, metabolites, lipidomics, ring trial, platform comparison, depression, mass spectrometry, nuclear magnetic resonance (NMR), liquid chromatography–mass spectrometry (LC−MS)

## Abstract

Metabolomics methods often encounter trade-offs between quantification accuracy and coverage, with truly comprehensive coverage only attainable through a multitude of complementary assays. Due to the lack of standardization and the variety of metabolomics assays, it is difficult to integrate datasets across studies or assays. To inform metabolomics platform selection, with a focus on posttraumatic stress disorder (PTSD), we review platform use and sample sizes in psychiatric metabolomics studies and then evaluate five prominent metabolomics platforms for coverage and performance, including intra-/inter-assay precision, accuracy, and linearity. We found performance was variable between metabolite classes, but comparable across targeted and untargeted approaches. Within all platforms, precision and accuracy were highly variable across classes, ranging from 0.9–63.2% (coefficient of variation) and 0.6–99.1% for accuracy to reference plasma. Several classes had high inter-assay variance, potentially impeding dissociation of a biological signal, including glycerophospholipids, organooxygen compounds, and fatty acids. Coverage was platform-specific and ranged from 16–70% of PTSD-associated metabolites. Non-overlapping coverage is challenging; however, benefits of applying multiple metabolomics technologies must be weighed against cost, biospecimen availability, platform-specific normative levels, and challenges in merging datasets. Our findings and open-access cross-platform dataset can inform platform selection and dataset integration based on platform-specific coverage breadth/overlap and metabolite-specific performance.

## 1. Introduction

Metabolomics can characterize the global biochemical activity of a biological entity as it is shaped by external factors including lifestyle and drug [1,2]. In parallel, the etiology of psychopathologies is shaped by complex interactions between intrinsic biological features and external factors, which is exemplified in posttraumatic stress disorder (PTSD) [3]. The temporal sensitivity of metabolomics is being leveraged to define environmental influences, pathological mechanisms, and drug-modified clinical states in psychiatric research [4,5]. Thus, metabolomics can provide valuable insights into the discovery of biological markers of disease states and advance precision medicine efforts through integration with other ‘omics [6,7]. This can be especially impactful for psychopathologies such as PTSD, which relies on subjective symptom reporting for diagnosis and has no biological markers approved by the U.S. Food and Drug Administration (FDA). Yet, metabolomic characterization of the disease is complicated by extensive variations in metabolomic techniques, including platform-specific metabolite coverage, detection of unknowns, precision and accuracy of measurements, and measurement variability over time, which can complicate longitudinal study designs [8]. Here, we took an empirical approach to identify the best fit-for-purpose metabolomics platform for characterizing pathological mechanisms or biological disease markers, with a focus on PTSD.

Metabolomics techniques are rapidly advancing, although highly variable. However, techniques can be organized into two main categories: (1) absolute quantitative (targeted) approaches and (2) relative quantitative (untargeted/discovery-based) approaches [9]. Targeted approaches enable absolute quantification of a defined set of metabolites, thereby facilitating longitudinal data collection; however, one inherent caveat is the narrowed scope of metabolite detection [10]. In contrast, discovery-based approaches can provide the most comprehensive metabolome coverage; however, present coverage from any single analytical platform is only a fraction of the 19,174 metabolites detected thus far in blood [11]. One strategy to overcome the limited coverage of any individual metabolomics assay is to combine datasets from different analytical platforms, but obstacles for merging datasets include the use of relative measurements rather than absolute quantitative measurements. For both discovery-based and targeted approaches, the size, type, and number of metabolites captured as well as measurement precision are shaped by sample preparation and analytical methods (i.e., metabolite extraction, separation, and ionization).

Studies comparing coverage and analytical performance across discovery-based and targeted metabolomics approaches have focused on metabolite coverage and intra-assay performance [8,12]. Virtually all platform comparisons report highly platform-specific metabolite coverage, with several citing the more expansive metabolite coverage of discovery-based approaches as an advantage [12,13]. Previous cross-platform comparisons have supported superior precision in targeted approaches [10], although metabolite measurements from targeted and untargeted platforms can be highly correlated [8,10,12,14,15]. To the best of our knowledge, the current study is the first evaluation of inter-assay performance across both discovery-based and targeted metabolomics approaches.

Here, we evaluated intra- and inter-assay performance in a longitudinal assessment of discovery and targeted commercial vendors that are capable of high-throughput, which is necessary for characterizing metabolomic signatures of heterogenous psychiatric disorders [16]. Given that discovery and targeted approaches differentially resolve methodological trade-offs to optimize distinct metrics, we leveraged several metrics relevant to biomarker discovery, including metabolite coverage and performance metrics in line with prior cross-platform comparisons [8,12]. To understand how to optimize metabolomics investigations in psychiatric research, we focused on PTSD as an exemplar of complex interactions between intrinsic biological features and external factors that precipitate psychopathologies [3]. We first mapped metabolites previously implicated in PTSD with a review of prior case vs. control metabolomics studies. Understanding the breadth of metabolomics efforts in PTSD enabled us to evaluate platform-specific coverage for PTSD-associated metabolites. In parallel, we evaluated the comprehensive platform-specific performance for all metabolites reported by each vendor. Finally, we considered the performance for PTSD-associated metabolites and limitations of the scope of metabolomics approaches applied thus far in PTSD. Moving forward, the cross-platform metabolomics database generated by this study can enable optimization of platform selection for other clinical states through mapping of performance for putative metabolites and weighing these details against coverage depth.

## 2. Results

### 2.1. Metabolites Affected in PTSD

**Figure 1:**
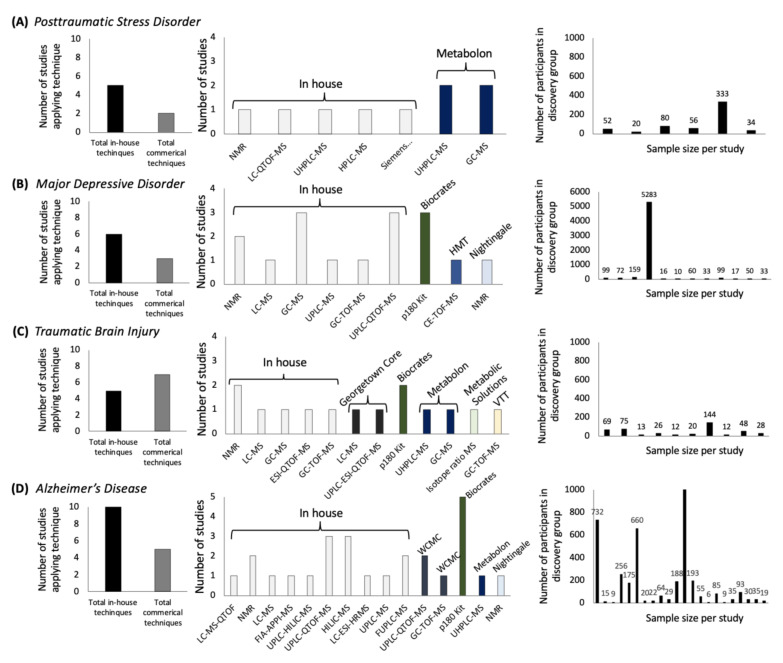
Metabolomics techniques and samples sizes in psychiatric research between 2015 and 2020 for four psychiatric conditions: (**A**) posttraumatic stress disorder, (**B**) major depressive disorder, (**C**) traumatic brain injury, and (**D**) Alzheimer’s disease.

To contextualize metabolomics efforts for PTSD in the broader landscape of psychiatric research, we mapped the use of metabolomics technologies for several psychopathologies (Figure 1; Supplementary Materials and Methods). Understanding the breadth of metabolomics techniques that have been applied to a disease state is informative because analytical methods dictate metabolite coverage, and previous efforts have found overlap in coverage across techniques as low as 27% [13], 15% [10], or 7% [12]. We found that evidence of metabolomic differences in published PTSD studies is accruing, but remains underexplored compared with other psychiatric conditions (Figure 1A,B,D). The breadth of techniques applied to PTSD have yielded coverage for only a fraction of the currently measurable portion of the human metabolome and samples size have been low compared with efforts in other psychiatric disease states. For studies of PTSD, exploratory group sizes have included 20 [17], 34 [18], 50 [19], 52 [20], and 77 [7] cases. In parallel, cross-cohort designs have been applied for other psychopathologies leveraging data from 5283 cases [21] or 22,623 participants [22]. A long-standing limitation for cross-cohort designs has been the complexity in the integration of metabolomics datasets generated through different approaches; although studies in PTSD have focused on discovery based approaches, a number of MS techniques have been utilized which suggests that the integration of currently available metabolomics data in PTSD would be challenging [23], but see also the integration of cross-cohort epigenetics with metabolomics in one cohort in [24].

To date, published case-control comparisons have provided valuable insights into the diverse metabolite classes that are likely to be relevant in PTSD (Supplementary Table S3). Classes with the highest count of implicated metabolites include amino acids, carnitines, essential fatty acids, glycerophospholipids, and glycerophosphocholines, with some discrepancy in the direction of effects for specific metabolites, i.e., citrate [7,17,19,20,25]. Currently, overlap in the findings across PTSD populations has been largely restricted to metabolite classes rather than individual metabolites, to the degree that even discovery and test groups recruited from the same hospital overlapped for only 2 metabolites out of a total of 33 implicated metabolites [26]. Additionally, most metabolomics studies in PTSD thus far have focused on case vs. control comparisons in male veterans, such that the role of sex and trauma type require further exploration [7,18,19,20,25] but see the efforts across sexes and trauma types in [17,24]. Another key consideration is that the emergence of PTSD requires trauma exposure; therefore, case vs. control studies that do not include longitudinal measurements, including assessments preceding trauma, cannot dissociate predisposing metabolomic features from consequences of trauma and PTSD etiology [24,27].

Low overlap across prior studies in metabolites associated with PTSD may reflect variation in population characteristics and methodologies used, including differences in fasting state, biofluid type and processing methods, time at collection, analytical technology, and data processing. Key methodological differences can be highlighted by comparing the landmark case vs. control study by Karabatsiakis et al. [17] with a more recent keystone metabolomics dataset used in multiple publications [7,25]. Karabatsiakis et al. [17] found 6 metabolite classes affected in individuals with PTSD (55% female; *n* = 20) compared with controls with varying degrees of trauma exposure (44% female; *n* = 18). In this study, participants were instructed to have regular breakfast and peripheral blood was collected at 10 am ± 15 min. Extracted serum was then analyzed by LC coupled to quadrupole/time-of-flight MS (LC-QToF-MS). By comparison, the second dataset is comprised of male combat-exposed veterans with PTSD (0% female; *n* = 83) or without PTSD (0% female; *n* = 82) [25]. Veterans reported to the laboratory under fasting conditions for peripheral blood collection at 8 am. Extracted plasma was then analyzed by Metabolon, Inc., using UHPLC-MS-MS optimized for basic metabolite species and GC-MS. Additional differences that are likely to shape the two datasets include the prevalence and type of comorbidities, the severity of the PTSD population, inclusion of ethnic minorities, use of internal standards, peak annotation methods, and data processing and normalization techniques [25,28,29]. Numerous metabolomics consortia and large-scale collaborative efforts have been convened to establish best practices in metabolomics including quality assurance and quality control practices [30], the use of standards [31,32], metabolite annotation confidence [33], as well as data processing and statistical approaches [34], and data reporting [35]. These important challenges are beyond the current scope, but it should be noted that fasting state and collection time appear to have differential effects across metabolite classes in both discovery-based and targeted approaches, and reproducibility is suggested to be lower in non-fasting samples as compared to fasting samples, such that methodological differences add complexity to the landscape of available PTSD metabolomics datasets [36,37,38].

Identifying the metabolomic signature of PTSD is likely to require future large-scale cohort studies or thoughtful integration of distinct cohorts on the same metabolomics platform, as has been done for depression [21] and Alzheimer’s Disease [22]. Direct comparison of distinct cohorts could help define population differences derived from factors previously demonstrated to shape PTSD symptomatology, including gender, trauma type (e.g., civilian vs. military trauma), early/cumulative trauma load, race/ethnicity, and education, as well as longitudinal studies to dissociate metabolomic features associated with risk vs. disease etiology [39,40].

### 2.2. Metabolite Coverage across Platforms

Metabolite coverage was highly variable across platforms (Table 1 and Table 2); reported metabolite classes were covered by an average of 1.8 vendors, with further vendor-specificity in the metabolites represented within each class. Several classes were represented by only one or few metabolites, emphasizing the expansiveness of the human metabolome, which is actively being charted and exceeds the scope of any metabolomics technology [11]. In addition to variation across metabolomics platforms, there was also variation within platforms across identical shipments (percent change in the number of metabolites between shipment 1 and shipment 2: Biocrates: −15.8%, HMT: 11.3%, Lipotype: 34.5%, Nightingale: 0%, Metabolon: 61.0% (discovery coverage only)). Metabolite classes consistently reported across both shipments are reported in Table 2.

**Figure 2:**
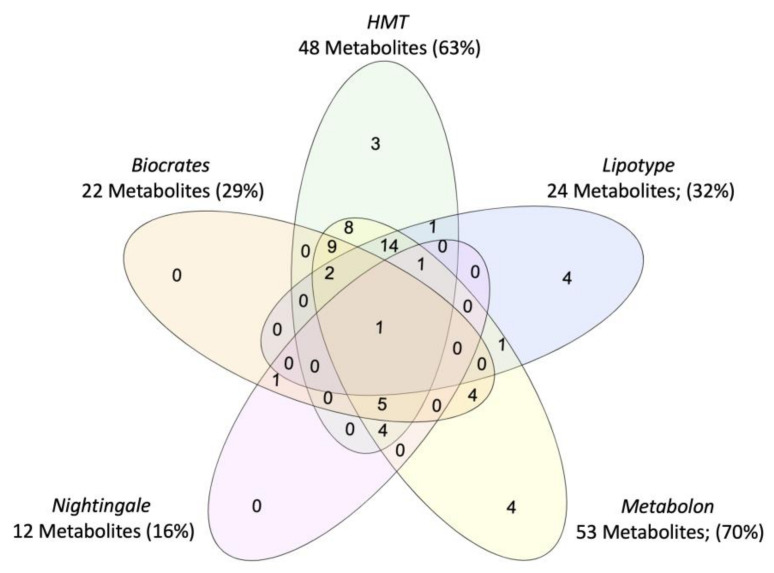
Venn diagram of coverage for PTSD-associated metabolites, for the five commercial metabolomics vendors assessed in the systematic platform comparison. Metabolites were identified through systematic review of case vs. control studies in PTSD.

**Figure 3:**
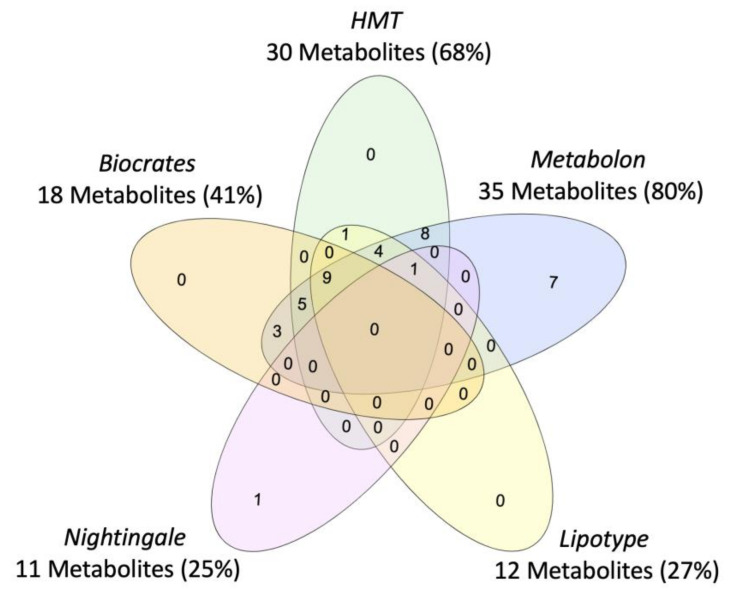
Venn diagram of coverage for metabolites associated with PARK2, for the five commercial metabolomics vendors assessed in the systematic platform comparison.

Vendor-specific coverage was determined for metabolites previously implicated in PTSD as a case-study (Figure 2), but review of metabolomics research in PTSD suggests that current findings are limited by the scope of the metabolome captured and the subpopulations represented (i.e., primarily male, combat trauma, European ancestry). Beyond case vs. control comparisons of the metabolome, efforts are ongoing to characterize the metabolomic signature of disease-linked genetic variants, which could represent a transdiagnostic approach. For example, metabolites associated with a *PARK2* variant have recently been mapped which may inform the biological mechanisms underpinning associations between *PARK2* and Parkinson’s disease, PTSD, diabetes mellitus, certain cancers, and inflammation ([41]; reviewed in [42]), see Figure 3. To note, tools to identify metabolites associated with biological pathways and genes of interest are constrained by currently available data and a field-wide need to identify unknowns [23].

### 2.3. Measurement Precision: Intra-Assay and Inter-Assay Coefficients of Variation

Metabolite measurement precision, assessed with technical replicates, was highly variable across and within metabolomics platforms (Table 1 and Table 2). Certain classes of metabolites were measured with high intra-assay precision (<10 CV%) by all platforms which measured metabolites in that class, (i.e., amino acids, hormones/steroids, and sphingomyelins). Conversely, some metabolite classes were measured with low intra-assay precision (>15% CV), in nearly all sample groups for all reporting vendors (i.e., diazines and fatty acyls). Similarly, certain metabolite classes were challenging to consistently detect and were reported in only one of the two samples shipment (i.e., diazines, glycerophospholipids, and keto acids and derivatives). Across all platforms, inter-assay precision was generally lower than intra-assay precision, and several metabolite classes had inter-assay CV%s greater than 30%. For example, the piperidine class had inter-assay CV%s of 53.4% and 63.2% but intra-assay CV%s of 14.8% and 16.3% for the PTSD and control group, respectively. The variability in precision across classes can be exemplified by comparing two classes of interest in PTSD, amino acids and fatty acids [19,20,25]. The amino acids class, which contains 10 metabolites implicated in PTSD, were measured with high to moderate intra- and inter-assay precision for all reporting vendors (4.8% to 14.2%). Conversely, for fatty acids, a class encompassing 11 metabolites implicated in PTSD across 4 studies [17,19,20,25], inter-assay precision ranged from 6.6% to 53.3% across all vendors and sample groups (with coverage differences across vendors). The substantial variability in precision across metabolite classes could derive from a myriad of challenges known to affect measurement quality in metabolomics, such as the frequency of isomers, fragmentation patterns, or ionization efficiency [14,43]. Given the substantial class-specificity in measurement precision, class-specific limitations in current technologies should be considered in the interpretation of available findings and prospective experimental designs to enable the dissociation of a biological signal.

### 2.4. Measurement Accuracy: Comparison to Known Values in NIST Reference Plasma

The accuracy of metabolite measurements, in comparison to NIST SRM 1950 pooled reference plasma reference values in the NIST certificate of analysis (COA), is provided in Table 3. Assessments of accuracy were constrained by (i) the fraction of classes represented in the NIST COA, (ii) vendor-specific coverage of metabolites, and (iii) the use of relative units which excluded Metabolon. Accuracy was evaluated for a set of amino acids listed in the NIST COA, which showed roughly similar high or moderate accuracy across plat-forms. Normalization methods informed by platform-specific normative levels could inform efforts to compare or merge datasets across metabolomics approaches. The majority of metabolites across all platforms were detected with excellent linearity across the dilution curve (i.e., coefficient of determination values near 1, suggesting that abundance is not a core obstacle in current metabolomics technologies; depicted in Supplementary Figure S1).

## 3. Discussion

Large-scale cohort studies are increasingly looking to advance multi-omics efforts through the integration of metabolomics, which can provide a summary of cellular activity as well as key insights into drug/toxin exposures [21,22,44]. Here, we evaluated five commercial metabolomics platforms that are frequently applied in large-scale metabolomics studies to characterize the state of current technologies. To do this, we evaluated metabolite coverage, measurement precision, and accuracy in a range of targeted and discovery metabolomics platforms including NMR and MS paired with direct infusion, LC, GC, and CE. This study advances ongoing efforts to evaluate assay performance across biomarker modalities, which has previously successfully identified highly sensitive platforms for measuring inflammatory cytokines [45].

Our findings demonstrated that metabolite coverage overlap across platforms was low, congruent with prior cross-platform comparisons [10,12,13]. Percentage overlap could not be determined due to irreconcilable differences in metabolite nomenclature and vendor-specific database harmonization. Platforms pioneering annotation can provide valuable living datasets; yet merging cohorts across approaches is likely to be impeded in the near future by challenges in marrying nomenclature and platform-specificity in the subspecies represented in a metabolite measurement. At the level of metabolite classes, an average of 1.8 vendors covered each reported class, with further vendor-specificity at the level of the metabolite. The maximum number of reported metabolites, reaching the 80% rule, was ~950, while the current estimated metabolite count in the human body is 114,100, with >19 k of these detected in blood [11]. While comprehensive coverage of the genome and transcriptome is currently possible, incomplete and platform-specific coverage in metabolomics is reasonable given the limited availability of metabolite standards and the extraordinary size and complexity of the human metabolome, which derives from endogenous sources as well as food, environment, microbes, drugs, etc. [10].

Platform-specific coverage is likely to be a challenge for diseases that are in early stages of characterizing mechanistic pathways and mapping putative metabolites as the set of metabolites implicated in a disease state are dependent upon the breadth of techniques that have been applied. For example, the metabolites currently implicated in PTSD can nearly be comprehensively covered by Metabolon (Supplementary Table S3). However, this is not surprising given that Metabolon’s technologies have been applied to PTSD populations [7,20,25], while platforms with distinct coverage, such as HMT, Lipotype, and Biocrates, have yet to be applied in large-scale case vs. control cohorts in PTSD, and could implicate novel metabolites based on their non-overlapping coverage. Given that researchers must weigh the benefits of applying multiple metabolomics technologies with cost, biospecimen availability, and challenges in merging datasets across approaches, there is great utility in vendors linking their internal libraries to prominent metabolite databases actively updated by the academic community. For example, HMDB links metabolites to published literature and key available information, including normative data, biological properties, and known associated diseases. In the future, harmonizing metabolite nomenclature is likely to increase in importance as those at the frontier expand annotation of the human metabolome.

Comparisons of precision and accuracy across absolute and relative quantitative metabolomics approaches must consider trade-offs in coverage and performance inherent in current technologies [8], As expected, the lowest coefficients of variation were yielded by NMR, with a trade-off in coverage breadth [15,46]. Lower precision was found in lipid classes across platforms, reflecting prior reports that quantification of lipids is difficult due to lipid solubility, incomplete separation during chromatography, and a variety of other challenges [47]. Overall, precision in the final normalized dataset was similar between targeted and untargeted MS approaches. This is consistent with a prior cross-platform comparison which reported that for overlapping metabolites the “quantitative results from the nontargeted assay are largely comparable to data derived from classical targeted assays” [13].

Conversely, within platforms, precision ranged from “low” to “high” across metabolite classes for all vendors, both within an assay and across assays. The variation in precision across metabolite classes likely reflects distinct features that can render certain classes consistently challenging to measure, including isomers, ionization efficiency, fragmentation, and the availability of standards [28,48]. For example, the NMR data varied from 0.88% CV for hydroxy acids to 27.57% CV for fatty acids, in the same plasma samples within the same assay. This degree of variation between metabolite classes was similar for discovery approaches. For example, Metabolon measured several metabolites classes with excellent precision (between 2–3% CVs) but measured amine oxides in duplicate PTSD samples with up to 45% CVs. Additionally, there were certain classes for which inter-assay drift was high across all vendors, for example, latosylceramides and glycerophospholipids were in the lowest precision category in both vendors that assessed these classes. The wide-spread variation in performance across metabolite classes indicates that researchers should weigh the strength of evidence for putative metabolites with the robustness of measurements for that class, and prospective studies should consider measurement robustness when determining sample size.

All metabolomics approaches shared greater inter-assay technical variation compared with intra-assay, including several classes which transition from “high” intra-assay precision to “low” inter-assay precision for some groups/vendors (acylcarnitines, diglycerides, bile acids, organooxygen compounds, etc.). Thus, longitudinal designs would benefit from the inclusion of blinded reference plasma within each assay run to anchor study-specific normalization, as well as maximizing the number of samples within each run (and minimizing batches) to the degree possible. Although prior findings have emphasized greater measurement drift for discovery approaches compared with targeted approaches, this gap appears to be attenuated in the current dataset, likely as a result of the rapidly advancing normalization and data processing procedures conducted by the vendors [10].

**Figure 4:**
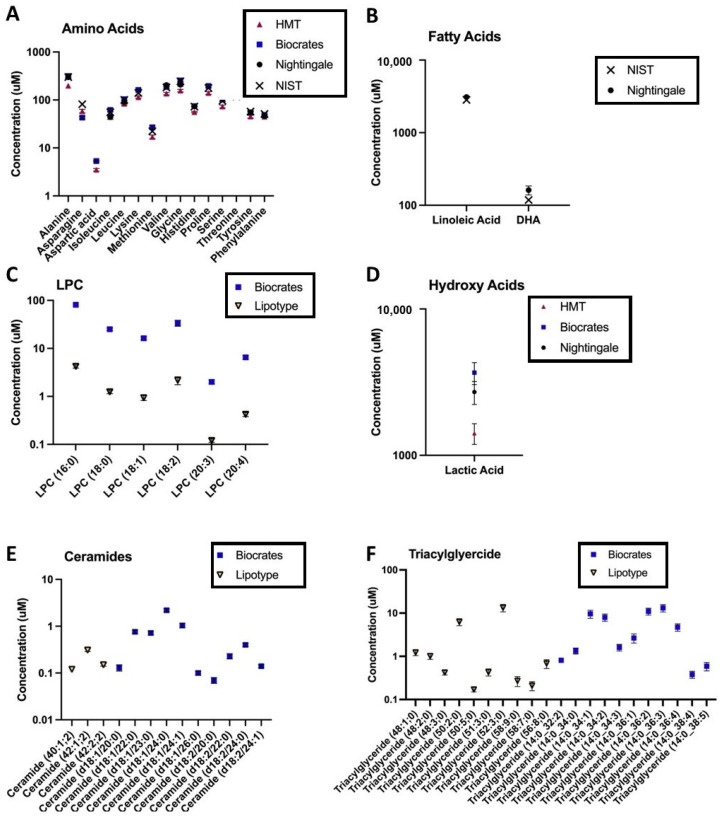
Platform-specific, log-transformed, average metabolite levels in control samples for vendors reporting absolute units; each point represents mean ± SEM for 11 control samples in total: 9 control samples from 6 individuals (with 3 technical replicates), and 2 NIST pooled reference plasma samples. Each panel depicts the range of covered metabolites, across all assays, for an exemplar metabolite class: (**A**) amino acids, (**B**) fatty acids, (**C**) lysophosphatidylcholines (LPC), (**D**) hydroxy acids, (**E**) ceramides, and (**F**) triglycerides. Depicted data are from the second sample shipment. NIST = concentrations reported in the National Institute of Standards and Technology (NIST) SRM 1950 Certificate of Analysis (COA, revised June 2020).

For certain metabolite classes, vendor reported measurements were consistently lower compared with concentrations in the NIST COA or values reported by other vendors (for example, Figure 4C). There are many possible explanations for systematically lower values in a metabolite or metabolite class: subspecies or isomers may not be captured by a specific approach, additionally fragmentation, matrix effects, and ion suppression can result in random or systematic errors [15]. These latter challenges may be exacerbated for direct infusion techniques which do not have elution order to aid in metabolite annotation [14,49]. Systematic differences in the reported concentration of a metabolite or metabolite class may be especially important in light of increasing efforts to combine cohorts to achieve the statistical power necessary for biomarker discovery in complex, heterogenous disease states [21,22]. Our findings and publicly available dataset provide a novel ability to address platform-specific “normative” levels. To enable the community to leverage the current cross-platform datasets for clinical states beyond PTSD, a platform exploration tool is being developed that will allow users to select specific metabolites of interest and explore coverage and technical variation between platforms. This platform exploration tool is currently undergoing beta-testing and will be launched in mid-2021. A representation of this visualization tool is provided in the Supplementary Materials.

## 4. Methods

### 4.1. Cross-Platform Comparison Design: Platforms Selected

Metabolomics platforms were selected to optimize the (i) diversity of analytical technologies, (ii) throughput, (iii) usage in large-scale and cross-cohort studies including psychiatric research, and (iv) capabilities of assays compatible with blood-based biofluids. Based on these considerations, we selected five metabolomics vendors: Biocrates (MxP^®^ Quant 500 kit), Human Metabolome Technologies (HMT) (Omega Scan using CE-Orbitrap, M-SCAN), Lipotype (Lipotype Shotgun Lipidomics), Metabolon (Global metabolomics), and Nightingale (Nightingale Blood Biomarker Analysis Service). All vendors analyzed duplicate plasma aliquots of parent samples in an identical, blinded, and randomized run order in their primary laboratory facilities (Biocrates: Innsbruck, Austria; HMT: Tsuruoka, Japan; Lipotype: Dresden, Germany; Metabolon: Morrisville, NC, USA; and Nightingale: Pittsburgh, PA, USA).

### 4.2. Cross-Platform Comparison Design: Clinical, Control, and Pooled Reference Plasma Samples

Each metabolomics vendor received two identical, blinded plasma shipments containing clinical samples, control samples, and pooled reference plasma (National Institute of Standards & Technology (NIST) Standard Reference Material (SRM) 1950). Each shipment contained 23 samples, identical across shipments, for a total of 46 samples. Metabolomics data from the control and PTSD samples were evaluated separately to account for potential population-specific performance. Given that identifying metabolites affected by PTSD was not the goal of the current study, and the current sample size is far from the scale necessary for the discovery of a disease signature, control and PTSD samples were not directly compared. A complete list of plasma samples included is provided in Supplementary Table S1.

To assess inter-assay variation, identical technical replicates were sent in two shipments; the first shipment was analyzed and data were received prior to sending the second shipment. Shipments were separated by 7–20 weeks pending vendor analysis timelines, with an average of 14 weeks between the two shipments. The sample run order was block randomized within 5 × 5 sample shipment boxes and was consistent across all platforms and shipments. All plasma technical replicates were aliquoted and prepared in parallel and shipped by the Indiana University Genetics Biobank (IUGB, Indianapolis, IN, USA). IUGB also generated a dilution curve of the NIST SRM 1950 diluted with physiological saline to 80%, 60%, and 40% of the starting concentration ([14]; additional detail in Supplementary Methods). To standardize preanalytical factors, all vendors received plasma aliquots shipped with dry ice, previously stored under the same conditions in blinded 500 uL aliquots and organized in an identical manner along with a sample manifest that provided the sample bar code, box name, sample position, and specimen type (human plasma). Vendors received plasma samples with an identical number of freeze/thaw cycles within each of the three sample types: PTSD, control, and NIST plasma samples. Vendors within the US received samples with overnight shipping. International vendors received samples through a carrier that monitors and maintains the levels of dry ice for sample integrity throughout the course of shipment. Shipment duration varied by international destination and was generally 2–5 days in the current context.

Use of human subjects was approved by the Stanford Institutional Review Board (IRB) under Protocol #25948. Clinical samples were obtained from 6 veterans with PTSD (3 male and 3 female), recruited through local VAs and assessed at Stanford University. PTSD status was determined by the Clinician-Administered PTSD Scale (CAPS). Blood was collected into vacutainer—K2 EDTA Purple-top tubes (10 mL) in the morning between 8–10 am, with participants instructed to be fasted overnight.

Control plasma samples were obtained by BioIVT and matched to clinical samples in terms of sex, age, time of day at collection, collection method (vacutainer), and overnight fasting condition (3 male and 3 female; details in Supplementary Table S2). Control blood was obtained with vacutainer—K2 EDTA Purple-top tubes with anticoagulants (6 mL; BD tube #: 367863) in the morning between 8–10 am, with participants instructed fast. Within 60 min, the whole blood was spun to obtain plasma at 1000–13,000× *g* for 10 min.

### 4.3. Metabolomics Analytical Platforms

**Figure 5:**
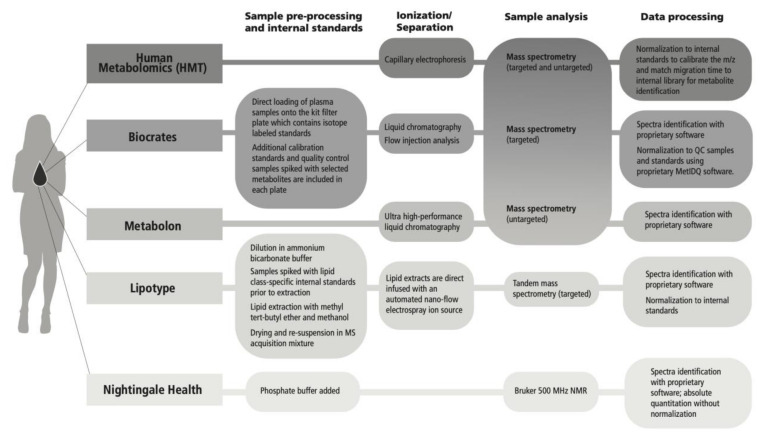
Metabolomics analytical methods across evaluated platforms.

Analytical technologies were mapped for the metabolomics platforms evaluated (Figure 5); approaches included nuclear magnetic resonance (NMR) and mass spectroscopy (MS) (discovery and targeted). For MS, a direct infusion technique was included as well as liquid chromatography (LC), gas chromatography (GC), and capillary electrophoresis (CE) separation techniques, and flow injection analysis (FIA). Metabolite coverage and the degree of quantitation varies across platforms based on inherent trade-offs that shape data output. For example, LC-MS is a versatile technique with a broad linear range, but it may be less precise compared with NMR, and LC-MS is blind to metabolites that do not ionize, which are detectable by techniques that do not require ionization [15]. Ionization efficiency varies across lipid classes as it has been shown to depend largely on the lipid head group, especially in direct infusion methods [14]. Direct infusion can enhance throughput but may cause suppression of low abundance species [50]. Ion suppression, particularly as a matrix effect, can also be an issue for LC-MS techniques if high abundance ions suppress the ionization of coeluting ions because of competition between ions [43]. NMR benefits from greater structural information that can aid in metabolite identification, but generally has lower sensitivity, therefore lower coverage, compared with MS based approaches [46]. Additionally, overlapping peaks from −CH, −CH2, −CH3 groups can increase quantitative error, despite the high accuracy of NMR [15]. Comprehensive descriptions of each analytical platform have been provided by the vendors, excluding proprietary data processing methods, and are located in the Supplementary Methods.

### 4.4. Metabolites Affected in Posttraumatic Stress Disorder (PTSD)

To understand the scope of metabolomics in psychiatric research and contextualize efforts in PTSD, we conducted a review of current literature to map (i) metabolomics techniques that have been applied and (ii) the number of participants (sample size) in discovery groups. We evaluated PTSD and three psychopathologies with overlapping symptomatology or frequent comorbidity with PTSD: major depressive disorder, traumatic brain injury, and Alzheimer’s disease. Metabolomics analytical techniques were categorized as commercial or “in house” academic assay approaches. For the “in house” metabolomics studies, authors did not specify vendor or core facility, such that it was presumed that procedures were run by the academic authors (authors were not contacted). A comprehensive search of the Google Scholar database was conducted for each disease state of interest using the following criteria for inclusion: (i) conducted in humans in the disease state of interest; (ii) published in a peer reviewed journal; (iii) assessed in a blood-based biofluid; (iv) an original report of empirical metabolomics data; and (v) published in or after 2015. Details extracted included: (1) number of clinical participants; (2) number of healthy control individuals (separated by discovery vs. test sets if applicable); (3) metabolomics analytical technology; and (4) vendor (if applicable). For PTSD, a search was conducted on 21 July 2020 using the following keywords: (“PTSD” OR “posttraumatic stress disorder”) AND “metabolomics”. A total of 100 studies were reviewed for inclusion, with a final total of 7 studies selected for inclusion. As PTSD was the focal disease state, this list was updated to include [26], which was published after the initial search date. For major depressive disorder, a search was conducted on 17 July 2020 using the following keywords: (“depression” OR “major depressive disorder”) AND “metabolomics”. A total of 100 studies were reviewed for inclusion, with a final 15 studies included. For traumatic brain injury, a search was conducted on 17 July 2020 using the following keywords: (“Alzheimer” OR “Alzheimer’s”) AND “metabolomics”. A total of 100 studies were reviewed for inclusion, with a final 10 studies included. For Alzheimer’s disease, a search was conducted on 20 July 2020 using the following keywords: (“traumatic brain injury”) AND “metabolomics”. A total of 100 studies were reviewed for inclusion, with 24 studies included. The full list of studies included is provided in the Supplementary Materials.

### 4.5. Metabolite Coverage and the Nomenclature across Platforms

To enable the assessment of the coverage overlap across different platforms, vendor reported metabolite nomenclature was collaboratively harmonized to Human Metabolome Database (HMDB) IDs for the identified metabolites, where possible [11]. In some cases, nomenclature could not be matched across vendors, particularly for some lipid classes and reported “unknowns”. Levels for unknown metabolites were provided by HMT and Metabolon; unknowns represent analytes in a vendor’s internal library which can be consistently measured and may be identified in the future, but currently do not have known identities. Unknowns are beyond the scope of the current effort; however, reported levels for unknowns are provided in the Supplementary Materials. Some vendors provided a list of subspecies and structural isomers represented in their metabolite measurements where possible/applicable. This is highly beneficial as platform-specific capturing of metabolite subspecies can result in platform-specific normative levels and can pose challenges for integrating datasets across approaches (Biocrates, Metabolon, HMT). Metabolite coverage for a platform can change over time as annotation algorithms are updated. Nightingale was the only vendor to report a change in their algorithms between shipments, reflecting an expansion of their internal dataset (~July, 2020). The Nightingale data reported here represents their most up-to-date algorithm (as of the date of publication); for reference, shipment 1 Nightingale data, analyzed with the current and prior algorithm, are provided in the Supplementary Materials. Notably, only metabolite/lipid coverage is addressed here, but Nightingale measures clinical analytes, which may be of great value beyond the current context (e.g., albumin, apolipoproteins, total omega-3 and omega-6 fatty acids, total cholesterol, and VLDL, LDL, HDL, HDL_2_, HDL_3_, esterified, as well as free cholesterol). To compare across platforms, coverage overlap was visualized with Venn diagrams [51].

### 4.6. Measurement Precision: Intra-Assay and Inter-Assay Coefficients of Variation

Duplicate aliquots from the same parent sample were included in two identical shipments. The difference between measurements of the duplicate technical replicates were determined for each annotated metabolite both within each shipment (( σ /(a_1_ + b_1_/2)) × 100) and across shipments, representing separate assay runs (( σ /(a_1_ + a_2_/2)) × 100) to determine percent coefficients of variation (CV%).

### 4.7. Measurement Accuracy: Comparison to Known Values in the National Institute of Standards and Technology (NIST) Reference Plasma

For the subset of metabolites which have concentrations reported in the NIST Certificate of Analysis (COA, revised June 2020), percent accuracy was determined for metabolites reported by each vendor in the NIST SRM 1950 samples ((reported value—NIST COA value)/NIST COA value) × 100; using shipment 1 data). For the dilution curve of the NIST reference plasma, containing samples at 100%, 80%, 60%, and 40% of the initial concentration, linearity was assessed by fitting a zero-intercept linear regression to the shipment 1 data in R version 4.0.4. The coefficient of determination (R^2^) was calculated as a goodness of fit parameter for each metabolite reported by each vendor, using all points in the dilution curve. If value(s) were missing for any NIST sample, linearity was not assessed for that metabolite reported by that vendor.

### 4.8. Data Analysis and Visualization

Missing data in metabolomics can result from analytical, computational, and biological factors [52,53]. Here, vendor-applied methods for missing data were not modified (i.e., if a vendor imputed missing data, the imputed data were used; if a vendor reported a datum as below threshold or missing it was omitted not imputed or substituted). For vendors that imputed data, a pre-imputation version of the dataset is included in the Supplementary Materials where possible. Substitution was not used here because the source of missingness likely differed between analytical platforms and therefore substitution could have introduced biases, e.g., distortion in data distribution or underestimation of the standard deviation of a variable or group [54]. However, it is important to note that missing values are often imputed in an experimental context with a strategy reflecting the probable source(s) of missingness in the metabolomics platform used [54,55]. Therefore, to avoid unreliable variables with a large proportion of missing values, in the absence of substitution methods, the “modified 80% rule” was used which states that a metabolite should be excluded if the proportion of non-missing elements account for less than 80% of the data for that metabolite in each biological group (control and PTSD in the current context) [54,56].

## 5. Conclusions

Our findings emphasize that comprehensive coverage is not yet possible for the metabolome and large-scale metabolomics platforms yield distinct coverage, with class-specific performance and measurement variability within and across all platforms. Therefore, researchers selecting a platform must weigh (i) the breadth and depth of coverage previously applied to their clinical state of interest, (ii) the strength of evidence for putative metabolites, and (iii) class-specific measurement robustness, which can be interrogated for metabolites of interest using the publicly available dataset generated by the current study. For diseases in nascent stages of metabolomics characterization, researchers may be able to leverage information on metabolites associated with genes or proteins of interest (e.g., [57] or metabolites associated with biological pathways (e.g., [58]. Numerous publicly available databases have been developed to support these efforts (e.g., Reactome, MetaCyc). Further efforts are underway to define species-specific reference values across biospecimen types, as well as the impact of clinical variables on population-level normative values including sex and age [11].

Beyond species-specific considerations, effects of technical variation—including variation from metabolite stability over time or preanalytical variables such as freeze-thaw cycles—could be minimized through the inclusion of blinded technical plasma in every assay run to enable study-specific normalization. Similarly, distributing groups across runs in block randomization could minimize the impacts of batch variation where possible. For example, in an innovative recent metabolomics study in PTSD conducted by Konjevod et al. [26], discovery and test cohorts “were analyzed under the same conditions, one year apart”. Technical variation across assays has the capacity to obfuscate biological differences and may have limited the metabolites validated in the test cohort to 2 metabolites out of the 33 initially implicated metabolites [26].

Studies combining cohorts to increase statistical power are promising but can necessitate integrating metabolomics results across approaches, which is hindered by (i) vendor-specific nomenclature, (ii) platform-specificity in the metabolite subspecies represented in a metabolite measurement, and (iii) platform-specific “normative” levels. The former two challenges may be ameliorated by harmonizing to a publicly available database where possible (e.g., HMDB). For the latter, strategies for integrating metabolomics datasets may be informed by our findings defining metabolite classes which are subject to systematically lower measurements in specific platforms (fatty acids, LPCs, etc.) or greater inter-assay variation for some or all platforms (glycerophospholipids, ceramides, quinolines, etc.).

## Figures and Tables

**Table 1:** Intra-assay Percent Coefficient of Variance (CV%) within Metabolite Classes and CV% Standard Deviation (SD) for Technical Replicates of PTSD and Control Samples in Shipment 1.

Intra-Assay Precision: Shipment 1																					
	PTSD Average CV%	Control Average CV%	Count	Range	SD	PTSD Average CV%	Control Average CV%	Count	SD	PTSD Average CV%	Control Average CV%	Count	SD	PTSD Average CV%	Control Average CV%	Count	SD	PTSD Average CV%	Control Average CV%	Count	SD
Metabolite Class	Biocrates					HMT				Nightingale				Lipotype				Metabolon			
Acylcarnitines	10.33	9.88	14	2.07–76.09	15.67	5.66	5.81	35	7.43									9.03	8.39	21	7.70
Amino Acids	3.53	8.12	40	0.87–19.01	3.14	6.85	6.93	22	2.56	2.78	6.01	9	3.76					8.84	9.56	52	8.22
Amino Acid Related	3.44	8.71		1.73–12.88	3.21																
Carboxylic Acids	3.85	7.20	2	2.70–8.02	2.25	5.10	5.62	8	2.83	2.99	6.66	3	2.38					11.32	9.13	101	13.59
Cholesteryl ester	8.46	14.69	18	3.42–48.13	8.44									4.23	13.30	13	6.84	4.63	4.00	26	2.20
Diglycerides	8.46	12.28	14	1.95–36.34	7.53									4.84	9.80	2	3.38	4.29	4.00	19	2.73
Diazines						21.00	18.45	3	21.16									15.39	13.46	5	12.60
Organonitrogen compounds						7.29	9.37	13	5.02									15.55	11.3	14	12.61
Purine nucleotides						9.69	16.16	5	9.32									14.18	12.48	10	8.09
Organooxygen compounds						12.03	9.63	6	7.35	1.04	6.44	2	4.07					9.19	9.90	6	8.38
Hydroxy acids and derivatives						4.04	6.79	5	2.47	0.88	3.05	1	NA					11.28	11.80	16	10.81
Keto acids and derivatives						1.62	5.52	3	2.24	4.29	26.20	2	18.87					8.35	8.18	13	4.94
Ceramides	6.53	8.60	25	0.59–29.33	5.33	11.36	10.43	5	4.62					5.03	6.77	2	3.06	7.22	7.10	11	5.55
Lactosylceramide						19.98	15.25	13	13.62									10.15	14.99	12	10.36
Glucosylceramide						21.84	19.55	13	11.15												
Dihexosylceramides	10.30	9.78	10	2.45–34.65	7.59																
Trihexosylceramides	14.16	17.94	6	4.69–43.82	11.60																
Dihydroceramide																		19.07	17.15	12	14.73
Hexosylceramide	9.56	11.32	19	1.33–27.20	5.81													6.28	8.33	12	4.34
Triglycerides	7.33	13.27	235	0.78–38.42	3.72									4.88	6.49	32	2.46	1.92	5.96	21	2.19
Hormones/Steroids	2.53	6.43	3	1.48–7.15	2.34	5.88	6.28	9	4.77									8.65	6.18	26	3.79
Fatty Acids	8.10	8.16	7	3.63–11.43	2.60	6.92	9.11	32	4.97	27.57	3.89	2	15.39					3.58	3.53	29	2.15
Fatty Acyls						28.92	29.75	4	19.76									18.17	13.20	83	12.09
Biogenic Amines	4.74	10.52	3	3.19–11.69	3.47																
Bile Acids	5.84	7.73	12	2.48–12.04	2.77	4.66	6.47	4	7.22									11.42	8.94	22	6.95
Indoles and Derivatives	3.36	10.40	3	2.69–14.21	4.56	6.51	7.37	1	NA									7.45	5.82	9	3.97
Lysophosphatidyl-cholines (LPC)	13.05	11.32	14	0.91–36.05	10.66	3.34	3.71	27	1.69					4.38	14.44	6	5.36	7.28	10.10	15	5.14
Glycerophosphocholines						7.09	7.76	19	10.79												
Phosphatidyl-cholines (PC)	7.98	9.78	73	1.78–67.84	9.56									7.96	11.15	63	5.22	8.12	5.98	18	4.44
Sphingomyelins	3.89	7.68	15	1.88–11.73	2.64									5.79	8.62	12	2.89	3.45	3.05	12	1.79
Sphingolipids						14.28	13.47	12	10.86									6.13	7.79	2	2.11
Sphinganine								6													
Sphingosine						11.76	12.89	6	9.48												
Glycerophospholipids						10.41	9.67	15	8.70									19.61	22.64	7	16.45
Glycerolipids (Monoacylglycerol)																		21.69	26.95	25	14.48
Carboximidic acids and derivatives						4.93	4.33	1	NA									15.21	17.34	4	10.22
lyso-Phosphatidylethanolamine (LPE)						10.36	8.40	22	7.95					8.83	15.24	9	5.24	5.18	6.38	8	4.88
Phosphatidylcholine (-ether) (LPC-O)														13.02	16.40	41	8.64				
Phosphatidylethanolamine (PE)														9.22	15.06	15	6.79	4.38	8.11	12	6.46
Phosphatidylethanolamine (-ether) (LPE-O)														11.54	15.20	16	7.76				
Phosphatidylinositol (LPI)						7.37	8.44	14	6.05					8.95	16.88	15	7.62	10.83	18.82	6	8.90
Lyso-Phosphatidylserine (LPS)						11.21	15.84	7	8.97												
Glycerophosphoglycerols (LPG)						9.23	10.92	14	6.71												
Vitamins and Cofactors	1.09	10.44	1		NA													2.37	5.87	1	NA
Alkaloids	4.69	13.74	1		NA													3.86	2.48	2	0.80
Amine (Oxides)	6.92	8.23	1		NA													45.46	12.49	1	NA
Carbohydrates and Related	2.50	6.33	1		NA													10.81	14.37	34	13.62
Cresols	2.07	7.14	1		NA																
Imidazopyrimidines						11.35	5.44	1	NA									10.32	7.60	17	8.68
5′-deoxyribonucleosides						19.82	18.62	1	NA									7.84	8.63	1	NA
Nucleoside and nucleotide analogues																		13.05	3.4	1	NA
Pyrimidine nucleosides						1.33	7.94	1	NA									9.24	9.59	7	7.33
Pyridines and derivatives																		6.69	7.90	10	5.76
Quinolines and derivatives																		11.85	13.64	3	7.42
Phenols																		9.22	4.79	3	5.40
Prenol lipids																		17.87	8.77	7	8.13
Imidazole ribonucleosides and ribonucleotides																		12.17	5.5	1	NA
Benzene and substituted derivatives																		12.72	9.49	14	10.36
Phenylpropanoic acids																		6.52	5.82	9	8.63
Tetrapyrroles and derivatives																		3.45	11.6	2	5.43
Cholesterol and derivatives																		7.53	9.5	2	4.40
Non-metal oxoanionic compounds																		2.94	3.17	2	1.81
Organic sulfuric acids and derivatives																		6.1	4.03	22	3.49
Organic sulfonic acids and derivatives																		2.47	5.59	2	3.48
Organic carbonic acids and derivatives																		6.33	9.48	2	3.88
Organic phosphoric acids and derivatives																		9.53	7.87	1	NA
Benzothiazepines																		2.29	6.17	2	5.83
Bilirubins																		9.03	5.41	2	6.66
Dihydrofurans																		6.94	3.07	2	3.68
Alkyl halides																		2.9	3.98	2	1.49
Sulfinic acids and derivatives																		11.13	16.04	1	NA
Azoles																		11.11	10.6	7	5.90
Azolidines																		4.34	6.92	1	NA
Cinnamic acids and derivatives																		4.07	13.92	1	NA
Peptidomimetics																		20.02	14.52	1	NA
Piperidines																		14.81	16.27	1	NA
Pyrrolidines																		4.09	5.48	1	NA
Coumarins and derivatives																		0	14.1	1	NA

Notes: “High” precision is shown in green (≤10%), “moderate” in yellow (10% < x < 20%), and “low” in red (≥20%).

**Table 2:** Inter-Assay percent Coefficient of Variance (CV%) within Metabolite Classes for Technical Replicates of PTSD and Control Samples across Shipment 1 and Shipment 2.

Inter-Assay Precision: Shipment 1 vs. Shipment 2															
	PTSD Average CV%	Control Average CV%	Standard deviation (SD)	PTSD Average CV%	Control Average CV%	Standard deviation (SD)	PTSD Average CV%	Control Average CV%	Standard deviation (SD)	PTSD Average CV%	Control Average CV%	Standard deviation (SD)	PTSD Average CV%	Control Average CV%	Standard deviation (SD)
Metabolite Class	Biocrates			HMT			Nightingale			Lipotype			Metabolon		
Acylcarnitines	7.21	11.48	2.78	12.21	12.10	8.64							15.27	13.52	9.93
Amino Acids	5.88	11.95	3.57	9.10	9.79	4.02	4.80	7.37	3.48				12.84	14.21	8.03
Amino Acid Related	7.63	13.90	6.86												
Carboxylic Acids	7.54	13.71	4.05	10.60	8.90	5.83	5.92	7.90	1.51				15.38	13.91	12.75
Cholesteryl ester	11.71	14.47	2.58							8.07	8.93	5.25	10.16	8.68	7.77
Diglycerides	17.42	22.27	10.62							9.13	13.25	6.27	10.97	10.06	4.53
Diazines				16.42	21.57	14.04							33.22	29.05	24.92
Organonitrogen compounds				13.31	11.50	8.24							14.25	13.66	7.67
Purine nucleotides				30.06	38.64	18.99							11.49	11.84	5.98
Organooxygen compounds				31.07	37.08	13.17	4.89	6.94	2.62				31.34	26.85	28.72
Hydroxy acids and derivatives				10.88	11.10	6.05	1.88	3.57	NA				17.76	17.90	12.03
Keto acids and derivatives				14.10	16.66	2.83	10.58	19.49	9.58				15.64	13.55	8.45
Ceramides	16.04	19.50	8.65	11.34	15.68	4.34				6.58	6.47	0.78	10.10	7.88	4.44
Lactosylceramide				15.32	16.79	8.06							16.61	17.51	13.28
Glucosylceramide				19.30	23.43	9.77									
Dihexosylceramides	11.99	17.82	5.18												
Trihexosylceramides	17.08	23.39	4.52												
Dihydroceramide													18.63	21.21	12.20
Hexosylceramide	14.23	17.72	6.01										11.44	10.57	3.49
Triglycerides	15.42	19.35	7.56							8.34	6.67	2.20	9.3	7.65	2.61
Hormones/Steroids	15.59	18.54	9.04	8.08	10.35	5.86							15.39	13.53	5.84
Fatty Acids	13.80	18.43	7.40	24.26	27.33	33.29	53.25	9.39	26.15				6.89	6.60	3.39
Fatty Acyls				4.21	4.99	NA							20.94	17.94	9.44
Biogenic Amines	6.37	18.78	10.04												
Bile Acids	21.36	23.51	28.05	5.99	10.49	10.09							21.36	20.83	11.05
Indoles and Derivatives	8.60	15.22	4.59										13.11	12.47	5.93
Lysophosphatidyl-cholines (LPC)	13.24	17.78	8.17	6.45	8.75	3.38				9.13	8.85	0.77	17.64	17.23	12.98
Glycerophosphocholines				6.56	7.21	2.76									
Phosphatidyl-cholines (PC)	10.11	14.43	6.97							10.24	13.18	10.69	13.65	13.98	13.68
Sphingomyelins	9.34	12.99	4.28							6.16	7.19	2.09	6.51	7.58	4.35
Sphingolipids				20.21	20.23	10.83							9.90	12.50	1.60
Sphinganine															
Sphingosine				19.34	19.49	10.01									
Glycerophospholipids				19.51	22.85	25.01							22.79	26.91	17.68
Glycerolipids (Monoacylglycerol)													35.32	34.69	15.53
Carboximidic acids and derivatives				23.26	3.55	NA							12.92	17.33	5.22
lyso-Phosphatidylethanolamine (LPE)				11.88	13.87	17.60				9.92	9.85	1.89	22.80	19.85	12.54
Phosphatidylcholine (-ether) (LPC-O)										14.35	15.00	6.39			
Phosphatidylethanolamine (PE)										12.50	14.70	6.08	12.16	12.16	8.38
Phosphatidylethanolamine (-ether) (LPE-O)										10.14	11.68	3.76			
Phosphatidylinositol (LPI)				9.81	10.24	6.62				13.27	13.27	7.59	39.03	49.61	11.64
Lyso-Phosphatidylserine (LPS)				19.00	20.64	7.74									
Glycerophosphoglycerols (LPG)				12.34	15.52	7.60									
Vitamins and Cofactors	7.59	13.77	NA										6.44	13.52	NA
Alkaloids													17.05	46.83	21.81
Amine (Oxides)	6.55	11.15	NA										28.84	24.42	NA
Carbohydrates and Related	6.61	13.25	NA										23.31	22.25	22.84
Cresols	4.39	11.88	NA												
Imidazopyrimidines													19.81	26.41	14.25
5′-deoxyribonucleosides				12.33	9.07	5.31							10.04	13.25	NA
Nucleoside and nucleotide analogues													44.29	15.88	NA
Pyrimidine nucleosides													21.49	19.06	15.04
Pyridines and derivatives													11.51	11.75	5.71
Quinolines and derivatives													20.42	20.85	13.19
Phenols													22.06	21.35	6.87
Prenol lipids													16.43	13.54	6.28
Imidazole ribonucleosides and ribonucleotides													7.24	7.26	NA
Benzene and substituted derivatives													23.37	23.26	13.29
Phenylpropanoic acids													17.47	23.77	12.32
Tetrapyrroles and derivatives													9.73	17.57	5.40
Cholesterol and derivatives													8.27	8.43	2.92
Non-metal oxoanionic compounds													4.07	4.04	0.41
Organic sulfuric acids and derivatives													23.64	25.3	27.37
Organic sulfonic acids and derivatives													7.08	10.9	6.10
Organic carbonic acids and derivatives													7.67	9	4.39
Organic phosphoric acids and derivatives													8.74	8.28	NA
Benzothiazepines													22.99	22.49	2.09
Bilirubins													10.37	13.28	NA
Dihydrofurans													10.09	6.84	2.42
Alkyl halides													7.17	5.97	2.77
Sulfinic acids and derivatives													9.57	21.87	NA
Azoles													17.19	12.88	7.62
Azolidines													14.11	17	NA
Cinnamic acids and derivatives													48.32	49.73	NA
Peptidomimetics													22.76	21.34	NA
Piperidines													53.36	63.22	NA
Pyrrolidines													10.55	13.2	NA
Coumarins and derivatives													30.57	17.89	NA

Notes: “High” precision is shown in green (≤10%), “moderate” in yellow (10% < x < 20%), and “low” in red (≥20%).

**Table 3:** Reporting Accuracy (%) compared with NIST Metabolites in Frozen Human Plasma (SRM 1950).

Accuracy (%)		Biocrates		HMT		Nightingale	
Analyte	NIST Value (uM)	Reported Value (uM)	Percent Difference	Reported Value (uM)	Percent Difference	Reported Value (uM)	Percent Difference
**Fatty Acids**							
C18:2 n-6 (Z,Z)-9,12-Octadecadienoic Acid (Linoleic Acid)	2838					2960	4.30%
C22:6 n-3. (Z,Z,Z,Z,Z,Z)-4,7,10,13,16,19-Docosahexaenoic Acid (DHA)	118					136	15.25%
**Amino Acids**							
Alanine	300	331	10.17%	211	−29.61%	312.246	4.08%
Glycine	245	288	17.72%	250	1.97%	240.87	−1.69%
Histidine	72.6	80	10.08%	59	−18.25%	70.0707	−3.48%
Isoleucine	55.5	66	18.92%	46	−17.02%	44.604	−19.63%
Leucine	100.4	114	13.05%	102	1.25%	87.7893	−12.56%
Lysine	140	151	7.73%	129	−7.60%		
Methionine	22.3	22	−0.94%	14	−38.50%		
Proline	177	199	12.30%	138	−22.19%		
Serine	95.9	98	2.14%	69	−27.57%		
Threonine	119.5	127	6.10%	92	−23.08%		
Tyrosine	57.3	61	6.48%	49	−13.95%	61.8318	7.91%
Valine	182.2	174	−4.40%	152	−16.30%	185.06	1.57%
Arginine	81.4	95	16.45%				
Cysteine	44.3	46	4.50%				
Cystine	7.8	8.0	2.76%				
Phenylalanine	51	57	12.54%	47	−8.27%	53.0223	3.97%
**Clinical Markers**							
Creatinine	60	65	7.69%	43	−28.14%	58.1642	−3.06%
Glucose	4560					4679.41	2.62%
Homocysteine	8.5	8.5	0.58%				
Cortisol	0.23	0.19	−17.92%				
Cholesterol	3917					3620	−7.58%

Notes: “High” accuracy is shown in green (≤10%), “moderate” in yellow (10% < x < 20%), and “low” in red (≥20%). Accuracy assessed only for vendors that reported quantitative units and not relative units; accuracy estimated using Shipment 1 data. All percent accuracy values are versus NIST COA values, such that a negative value is below the NIST-provided reference value.

## Data Availability

Data and the Metabolomics Platform Exploration Tool will be made available in the BRAIN Commons, a cloud-based platform for computational discovery designed for the brain health community at https://www.braincommons.org/publications/doi-10-3390-metabo-11090609/ accessed on 28 September 2022.

## Supplementary Materials

The following are available online at https://www.mdpi.com/2218-1989/11/9/609/s1, Figure S1: Linearity across the dilution curve of NIST pooled reference plasma (SRM 1950), Table S1: Metabolites and lipids implicated in posttraumatic stress disorder (PTSD) in previously published metabolomics studies, Table S2: List of samples sent to each metabolomics vendor in two identical shipments, Table S3: Metabolomics bake-off sample information.

## Definitions

*Analytical platform:* Refers to the entire methodology implemented by a commercial metabolomics vendor from receipt of samples to finalization of datasets, including sample preparation, standard use, analytical technology, and all proprietary metabolite identification and data processing methods [12]. *Coverage:* A metabolite was labeled “covered” according to the “modified 80% rule” which states that a metabolite should be excluded if the proportion of non-missing elements account for >80% of that metabolite’s data within each biological group [54,56]. In the current context, a metabolite was “covered” if it was reported by a vendor with no more than 20% of missing values per treatment group (control and PTSD), without additional substitution and imputation beyond that used by the vendors. *Technical replicate:* Plasma samples from control individuals and PTSD patients were separated into multiple identical 500 uL aliquots [14]. These identical aliquots were repeatedly measured by blinded metabolomics vendors both within the same assay run and across two different shipments, sent several weeks apart, to represent separate assay runs. *Intra-assay precision:* The percent coefficient of variation (CV%), for each annotated metabolite, between duplicate aliquots of identical parent samples within an assay run (( σ /(a_1_ + b_1_/2)) × 100) [8]. “High” precision was defined as ≤10%, “moderate” as 10% < x < 15%, “low” as ≥15%. ( σ = standard deviation of duplicate metabolite measurements within a vendor; a = vendor metabolite measurement for the first identical aliquot; b = vendor metabolite measurement for the second identical aliquot). *Inter-assay precision:* The percent coefficient of variation (CV%), for each annotated metabolite, between duplicate aliquots of identical parent samples across shipments, representing separate assay runs (( σ /(a_1_ + a_2_/2)) × 100). “High” precision was defined as ≤10%, “moderate” as 10% < x <15%, “low” as ≥15%. *Accuracy:* Percent deviation from “known” values, for the subset of metabolites that have concentrations reported in the NIST SRM 1950 Certificate of Analysis (COA, revised June 2020), calculated as (reported value—NIST COA value)/NIST COA value × 100) [8]. “High” accuracy was defined as ≤10%, “moderate” as 10% < x < 20%, “low” as ≥20%.
