# Correction: Chaby et al. Cross-Platform Evaluation of Commercially Targeted and Untargeted Metabolomics Approaches to Optimize the Investigation of Psychiatric Disease. *Metabolites* 2021, *11*, 609

**DOI:** 10.3390/metabo13080933

**Published:** 2023-08-09

**Authors:** Lauren E. Chaby, Heather C. Lasseter, Kévin Contrepois, Reza M. Salek, Christoph W. Turck, Andrew Thompson, Timothy Vaughan, Magali Haas, Andreas Jeromin

**Affiliations:** 1 Cohen Veterans Bioscience, New York, NY 10018, USA; lauren.chaby@cohenbio.org (L.E.C.); lasseterh@gmail.com (H.C.L.); andrew.thompson@cohenbio.org (A.T.); timothy.vaughan@cohenbio.org (T.V.); magali.haas@cohenbio.org (M.H.); 2 Department of Genetics, Stanford University School of Medicine, Stanford, CA 94305, USA; kcontrep@stanford.edu; 3 International Agency for Research on Cancer, Nutrition and Metabolism Branch, World Health Organisation, 150 Cours Albert Thomas, CEDEX 08, 69372 Lyon, France; r7salek@gmail.com; 4 Max Planck Institute of Psychiatry, Proteomics and Biomarkers, 80804 Munich, Germany; turck@psych.mpg.de

## Figure Legend

In the original publication [1], there was a mistake in the legend for Figure 4, a log scale for the axis has been applied to each graph to better show the included data points.

The correct legend appears below:

**Figure 4.** Platform-specific, log-transformed, average metabolite levels in control samples for vendors reporting absolute units; each point represents mean ± SEM for 11 control samples in total: 9 control samples from 6 individuals (with 3 technical replicates), and 2 NIST pooled reference plasma samples. Each panel depicts the range of covered metabolites, across all assays, for an exemplar metabolite class: (A) amino acids, (B) fatty acids, (C) lysophosphatidylcholines (LPC), (D) hydroxy acids, (E) ceramides, and (F) triglycerides. Depicted data are from the second sample shipment. NIST = concentrations reported in the National Institute of Standards and Technology (NIST) SRM 1950 Certificate of Analysis (COA, revised June 2020).

## Error in Figure/Table

In the original publication, there was a mistake in: 

1. Table 1, Table 2 and Table 3. The color coding in these tables does not match the footnote. This has been corrected.

2. Table 3. The amino acid, alanine, was omitted from the published manuscript. This is now included in the corrected table.

3. Table 3 as published. NIST standards were used for part of the study to compare the accuracy of the different measurements. In the measurement of fatty acids and cholesterol, the NIST standards reflect the total concentration of fatty acids and cholesterol. The vendors in the published manuscript were reporting free fatty acids and free cholesterol, and therefore the comparison represented in Table 3 is not valid. This discrepancy was brought to our attention after the paper was published. The fatty acid and cholesterol values and percent accuracy for Biocrates and Lipotype were removed.

4. Figure 4. Reflecting the updates from Table 3, Figure 4b has been edited and a log scale applied for all graphs in the figure to better show the included data points. 

The corrected Table 1, Table 2 and Table 3 and Figure 4 appear below:

**Table 1:** Intra-assay Percent Coefficient of Variance (CV%) within Metabolite Classes and CV% Standard Deviation (SD) for Technical Replicates of PTSD and Control Samples in Shipment 1.

Intra-Assay Precision: Shipment 1																					
	PTSD Average CV%	Control Average CV%	Count	Range	SD	PTSD Average CV%	Control Average CV%	Count	SD	PTSD Average CV%	Control Average CV%	Count	SD	PTSD Average CV%	Control Average CV%	Count	SD	PTSD Average CV%	Control Average CV%	Count	SD
Metabolite Class	Biocrates					HMT				Nightingale				Lipotype				Metabolon			
Acylcarnitines	10.33	9.88	14	2.07–76.09	15.67	5.66	5.81	35	7.43									9.03	8.39	21	7.70
Amino Acids	3.53	8.12	40	0.87–19.01	3.14	6.85	6.93	22	2.56	2.78	6.01	9	3.76					8.84	9.56	52	8.22
Amino Acid Related	3.44	8.71		1.73–12.88	3.21																
Carboxylic Acids	3.85	7.20	2	2.70–8.02	2.25	5.10	5.62	8	2.83	2.99	6.66	3	2.38					11.32	9.13	101	13.59
Cholesteryl ester	8.46	14.69	18	3.42–48.13	8.44									4.23	13.30	13	6.84	4.63	4.00	26	2.20
Diglycerides	8.46	12.28	14	1.95–36.34	7.53									4.84	9.80	2	3.38	4.29	4.00	19	2.73
Diazines						21.00	18.45	3	21.16									15.39	13.46	5	12.60
Organonitrogen compounds						7.29	9.37	13	5.02									15.55	11.3	14	12.61
Purine nucleotides						9.69	16.16	5	9.32									14.18	12.48	10	8.09
Organooxygen compounds						12.03	9.63	6	7.35	1.04	6.44	2	4.07					9.19	9.90	6	8.38
Hydroxy acids and derivatives						4.04	6.79	5	2.47	0.88	3.05	1	NA					11.28	11.80	16	10.81
Keto acids and derivatives						1.62	5.52	3	2.24	4.29	26.20	2	18.87					8.35	8.18	13	4.94
Ceramides	6.53	8.60	25	0.59–29.33	5.33	11.36	10.43	5	4.62					5.03	6.77	2	3.06	7.22	7.10	11	5.55
Lactosylceramide						19.98	15.25	13	13.62									10.15	14.99	12	10.36
Glucosylceramide						21.84	19.55	13	11.15												
Dihexosylceramides	10.30	9.78	10	2.45–34.65	7.59																
Trihexosylceramides	14.16	17.94	6	4.69–43.82	11.60																
Dihydroceramide																		19.07	17.15	12	14.73
Hexosylceramide	9.56	11.32	19	1.33–27.20	5.81													6.28	8.33	12	4.34
Triglycerides	7.33	13.27	235	0.78–38.42	3.72									4.88	6.49	32	2.46	1.92	5.96	21	2.19
Hormones/Steroids	2.53	6.43	3	1.48–7.15	2.34	5.88	6.28	9	4.77									8.65	6.18	26	3.79
Fatty Acids	8.10	8.16	7	3.63–11.43	2.60	6.92	9.11	32	4.97	27.57	3.89	2	15.39					3.58	3.53	29	2.15
Fatty Acyls						28.92	29.75	4	19.76									18.17	13.20	83	12.09
Biogenic Amines	4.74	10.52	3	3.19–11.69	3.47																
Bile Acids	5.84	7.73	12	2.48–12.04	2.77	4.66	6.47	4	7.22									11.42	8.94	22	6.95
Indoles and Derivatives	3.36	10.40	3	2.69–14.21	4.56	6.51	7.37	1	NA									7.45	5.82	9	3.97
Lysophosphatidyl-cholines (LPC)	13.05	11.32	14	0.91–36.05	10.66	3.34	3.71	27	1.69					4.38	14.44	6	5.36	7.28	10.10	15	5.14
Glycerophosphocholines						7.09	7.76	19	10.79												
Phosphatidyl-cholines (PC)	7.98	9.78	73	1.78–67.84	9.56									7.96	11.15	63	5.22	8.12	5.98	18	4.44
Sphingomyelins	3.89	7.68	15	1.88–11.73	2.64									5.79	8.62	12	2.89	3.45	3.05	12	1.79
Sphingolipids						14.28	13.47	12	10.86									6.13	7.79	2	2.11
Sphinganine								6													
Sphingosine						11.76	12.89	6	9.48												
Glycerophospholipids						10.41	9.67	15	8.70									19.61	22.64	7	16.45
Glycerolipids (Monoacylglycerol)																		21.69	26.95	25	14.48
Carboximidic acids and derivatives						4.93	4.33	1	NA									15.21	17.34	4	10.22
lyso-Phosphatidylethanolamine (LPE)						10.36	8.40	22	7.95					8.83	15.24	9	5.24	5.18	6.38	8	4.88
Phosphatidylcholine (-ether) (LPC-O)														13.02	16.40	41	8.64				
Phosphatidylethanolamine (PE)														9.22	15.06	15	6.79	4.38	8.11	12	6.46
Phosphatidylethanolamine (-ether) (LPE-O)														11.54	15.20	16	7.76				
Phosphatidylinositol (LPI)						7.37	8.44	14	6.05					8.95	16.88	15	7.62	10.83	18.82	6	8.90
Lyso-Phosphatidylserine (LPS)						11.21	15.84	7	8.97												
Glycerophosphoglycerols (LPG)						9.23	10.92	14	6.71												
Vitamins and Cofactors	1.09	10.44	1		NA													2.37	5.87	1	NA
Alkaloids	4.69	13.74	1		NA													3.86	2.48	2	0.80
Amine (Oxides)	6.92	8.23	1		NA													45.46	12.49	1	NA
Carbohydrates and Related	2.50	6.33	1		NA													10.81	14.37	34	13.62
Cresols	2.07	7.14	1		NA																
Imidazopyrimidines						11.35	5.44	1	NA									10.32	7.60	17	8.68
5′-deoxyribonucleosides						19.82	18.62	1	NA									7.84	8.63	1	NA
Nucleoside and nucleotide analogues																		13.05	3.4	1	NA
Pyrimidine nucleosides						1.33	7.94	1	NA									9.24	9.59	7	7.33
Pyridines and derivatives																		6.69	7.90	10	5.76
Quinolines and derivatives																		11.85	13.64	3	7.42
Phenols																		9.22	4.79	3	5.40
Prenol lipids																		17.87	8.77	7	8.13
Imidazole ribonucleosides and ribonucleotides																		12.17	5.5	1	NA
Benzene and substituted derivatives																		12.72	9.49	14	10.36
Phenylpropanoic acids																		6.52	5.82	9	8.63
Tetrapyrroles and derivatives																		3.45	11.6	2	5.43
Cholesterol and derivatives																		7.53	9.5	2	4.40
Non-metal oxoanionic compounds																		2.94	3.17	2	1.81
Organic sulfuric acids and derivatives																		6.1	4.03	22	3.49
Organic sulfonic acids and derivatives																		2.47	5.59	2	3.48
Organic carbonic acids and derivatives																		6.33	9.48	2	3.88
Organic phosphoric acids and derivatives																		9.53	7.87	1	NA
Benzothiazepines																		2.29	6.17	2	5.83
Bilirubins																		9.03	5.41	2	6.66
Dihydrofurans																		6.94	3.07	2	3.68
Alkyl halides																		2.9	3.98	2	1.49
Sulfinic acids and derivatives																		11.13	16.04	1	NA
Azoles																		11.11	10.6	7	5.90
Azolidines																		4.34	6.92	1	NA
Cinnamic acids and derivatives																		4.07	13.92	1	NA
Peptidomimetics																		20.02	14.52	1	NA
Piperidines																		14.81	16.27	1	NA
Pyrrolidines																		4.09	5.48	1	NA
Coumarins and derivatives																		0	14.1	1	NA

Notes: “High” precision is shown in green (≤10%), “moderate” in yellow (10% < x < 20%), and “low” in red (≥20%).

**Table 2:** Inter-Assay percent Coefficient of Variance (CV%) within Metabolite Classes for Technical Replicates of PTSD and Control Samples across Shipment 1 and Shipment 2.

Inter-assay Precision: Shipment 1 vs. Shipment 2															
	PTSD Average CV%	Control Average CV%	Standard deviation (SD)	PTSD Average CV%	Control Average CV%	Standard deviation (SD)	PTSD Average CV%	Control Average CV%	Standard deviation (SD)	PTSD Average CV%	Control Average CV%	Standard deviation (SD)	PTSD Average CV%	Control Average CV%	Standard deviation (SD)
Metabolite Class	Biocrates			HMT			Nightingale			Lipotype			Metabolon		
Acylcarnitines	7.21	11.48	2.78	12.21	12.10	8.64							15.27	13.52	9.93
Amino Acids	5.88	11.95	3.57	9.10	9.79	4.02	4.80	7.37	3.48				12.84	14.21	8.03
Amino Acid Related	7.63	13.90	6.86												
Carboxylic Acids	7.54	13.71	4.05	10.60	8.90	5.83	5.92	7.90	1.51				15.38	13.91	12.75
Cholesteryl ester	11.71	14.47	2.58							8.07	8.93	5.25	10.16	8.68	7.77
Diglycerides	17.42	22.27	10.62							9.13	13.25	6.27	10.97	10.06	4.53
Diazines				16.42	21.57	14.04							33.22	29.05	24.92
Organonitrogen compounds				13.31	11.50	8.24							14.25	13.66	7.67
Purine nucleotides				30.06	38.64	18.99							11.49	11.84	5.98
Organooxygen compounds				31.07	37.08	13.17	4.89	6.94	2.62				31.34	26.85	28.72
Hydroxy acids and derivatives				10.88	11.10	6.05	1.88	3.57	NA				17.76	17.90	12.03
Keto acids and derivatives				14.10	16.66	2.83	10.58	19.49	9.58				15.64	13.55	8.45
Ceramides	16.04	19.50	8.65	11.34	15.68	4.34				6.58	6.47	0.78	10.10	7.88	4.44
Lactosylceramide				15.32	16.79	8.06							16.61	17.51	13.28
Glucosylceramide				19.30	23.43	9.77									
Dihexosylceramides	11.99	17.82	5.18												
Trihexosylceramides	17.08	23.39	4.52												
Dihydroceramide													18.63	21.21	12.20
Hexosylceramide	14.23	17.72	6.01										11.44	10.57	3.49
Triglycerides	15.42	19.35	7.56							8.34	6.67	2.20	9.3	7.65	2.61
Hormones/Steroids	15.59	18.54	9.04	8.08	10.35	5.86							15.39	13.53	5.84
Fatty Acids	13.80	18.43	7.40	24.26	27.33	33.29	53.25	9.39	26.15				6.89	6.60	3.39
Fatty Acyls				4.21	4.99	NA							20.94	17.94	9.44
Biogenic Amines	6.37	18.78	10.04												
Bile Acids	21.36	23.51	28.05	5.99	10.49	10.09							21.36	20.83	11.05
Indoles and Derivatives	8.60	15.22	4.59										13.11	12.47	5.93
Lysophosphatidyl-cholines (LPC)	13.24	17.78	8.17	6.45	8.75	3.38				9.13	8.85	0.77	17.64	17.23	12.98
Glycerophosphocholines				6.56	7.21	2.76									
Phosphatidyl-cholines (PC)	10.11	14.43	6.97							10.24	13.18	10.69	13.65	13.98	13.68
Sphingomyelins	9.34	12.99	4.28							6.16	7.19	2.09	6.51	7.58	4.35
Sphingolipids				20.21	20.23	10.83							9.90	12.50	1.60
Sphinganine															
Sphingosine				19.34	19.49	10.01									
Glycerophospholipids				19.51	22.85	25.01							22.79	26.91	17.68
Glycerolipids (Monoacylglycerol)													35.32	34.69	15.53
Carboximidic acids and derivatives				23.26	3.55	NA							12.92	17.33	5.22
lyso-Phosphatidylethanolamine (LPE)				11.88	13.87	17.60				9.92	9.85	1.89	22.80	19.85	12.54
Phosphatidylcholine (-ether) (LPC-O)										14.35	15.00	6.39			
Phosphatidylethanolamine (PE)										12.50	14.70	6.08	12.16	12.16	8.38
Phosphatidylethanolamine (-ether) (LPE-O)										10.14	11.68	3.76			
Phosphatidylinositol (LPI)				9.81	10.24	6.62				13.27	13.27	7.59	39.03	49.61	11.64
Lyso-Phosphatidylserine (LPS)				19.00	20.64	7.74									
Glycerophosphoglycerols (LPG)				12.34	15.52	7.60									
Vitamins and Cofactors	7.59	13.77	NA										6.44	13.52	NA
Alkaloids													17.05	46.83	21.81
Amine (Oxides)	6.55	11.15	NA										28.84	24.42	NA
Carbohydrates and Related	6.61	13.25	NA										23.31	22.25	22.84
Cresols	4.39	11.88	NA												
Imidazopyrimidines													19.81	26.41	14.25
5′-deoxyribonucleosides				12.33	9.07	5.31							10.04	13.25	NA
Nucleoside and nucleotide analogues													44.29	15.88	NA
Pyrimidine nucleosides													21.49	19.06	15.04
Pyridines and derivatives													11.51	11.75	5.71
Quinolines and derivatives													20.42	20.85	13.19
Phenols													22.06	21.35	6.87
Prenol lipids													16.43	13.54	6.28
Imidazole ribonucleosides and ribonucleotides													7.24	7.26	NA
Benzene and substituted derivatives													23.37	23.26	13.29
Phenylpropanoic acids													17.47	23.77	12.32
Tetrapyrroles and derivatives													9.73	17.57	5.40
Cholesterol and derivatives													8.27	8.43	2.92
Non-metal oxoanionic compounds													4.07	4.04	0.41
Organic sulfuric acids and derivatives													23.64	25.3	27.37
Organic sulfonic acids and derivatives													7.08	10.9	6.10
Organic carbonic acids and derivatives													7.67	9	4.39
Organic phosphoric acids and derivatives													8.74	8.28	NA
Benzothiazepines													22.99	22.49	2.09
Bilirubins													10.37	13.28	NA
Dihydrofurans													10.09	6.84	2.42
Alkyl halides													7.17	5.97	2.77
Sulfinic acids and derivatives													9.57	21.87	NA
Azoles													17.19	12.88	7.62
Azolidines													14.11	17	NA
Cinnamic acids and derivatives													48.32	49.73	NA
Peptidomimetics													22.76	21.34	NA
Piperidines													53.36	63.22	NA
Pyrrolidines													10.55	13.2	NA
Coumarins and derivatives													30.57	17.89	NA

Notes: “High” precision is shown in green (≤10%), “moderate” in yellow (10% < x < 20%), and “low” in red (≥20%).

**Table 3:** Reporting Accuracy (%) compared with NIST Metabolites in Frozen Human Plasma (SRM 1950).

Accuracy (%)		Biocrates		HMT		Nightingale	
Analyte	NIST Value (uM)	Reported Value (uM)	Percent Difference	Reported Value (uM)	Percent Difference	Reported Value (uM)	Percent Difference
**Fatty Acids**							
C18:2 n-6 (Z,Z)-9,12-Octadecadienoic Acid (Linoleic Acid)	2838					2960	4.30%
C22:6 n-3. (Z,Z,Z,Z,Z,Z)-4,7,10,13,16,19-Docosahexaenoic Acid (DHA)	118					136	15.25%
**Amino Acids**							
Alanine	300	331	10.17%	211	−29.61%	312.246	4.08%
Glycine	245	288	17.72%	250	1.97%	240.87	−1.69%
Histidine	72.6	80	10.08%	59	−18.25%	70.0707	−3.48%
Isoleucine	55.5	66	18.92%	46	−17.02%	44.604	−19.63%
Leucine	100.4	114	13.05%	102	1.25%	87.7893	−12.56%
Lysine	140	151	7.73%	129	−7.60%		
Methionine	22.3	22	−0.94%	14	−38.50%		
Proline	177	199	12.30%	138	−22.19%		
Serine	95.9	98	2.14%	69	−27.57%		
Threonine	119.5	127	6.10%	92	−23.08%		
Tyrosine	57.3	61	6.48%	49	−13.95%	61.8318	7.91%
Valine	182.2	174	−4.40%	152	−16.30%	185.06	1.57%
Arginine	81.4	95	16.45%				
Cysteine	44.3	46	4.50%				
Cystine	7.8	8.0	2.76%				
Phenylalanine	51	57	12.54%	47	−8.27%	53.0223	3.97%
**Clinical Markers**							
Creatinine	60	65	7.69%	43	−28.14%	58.1642	−3.06%
Glucose	4560					4679.41	2.62%
Homocysteine	8.5	8.5	0.58%				
Cortisol	0.23	0.19	−17.92%				
Cholesterol	3917					3620	−7.58%

Notes: “High” accuracy is shown in green (≤10%), “moderate” in yellow (10% < x < 20%), and “low” in red (≥20%). Accuracy assessed only for vendors that reported quantitative units and not relative units; accuracy estimated using Shipment 1 data. All percent accuracy values are versus NIST COA values, such that a negative value is below the NIST-provided reference value.

**Figure 4:**
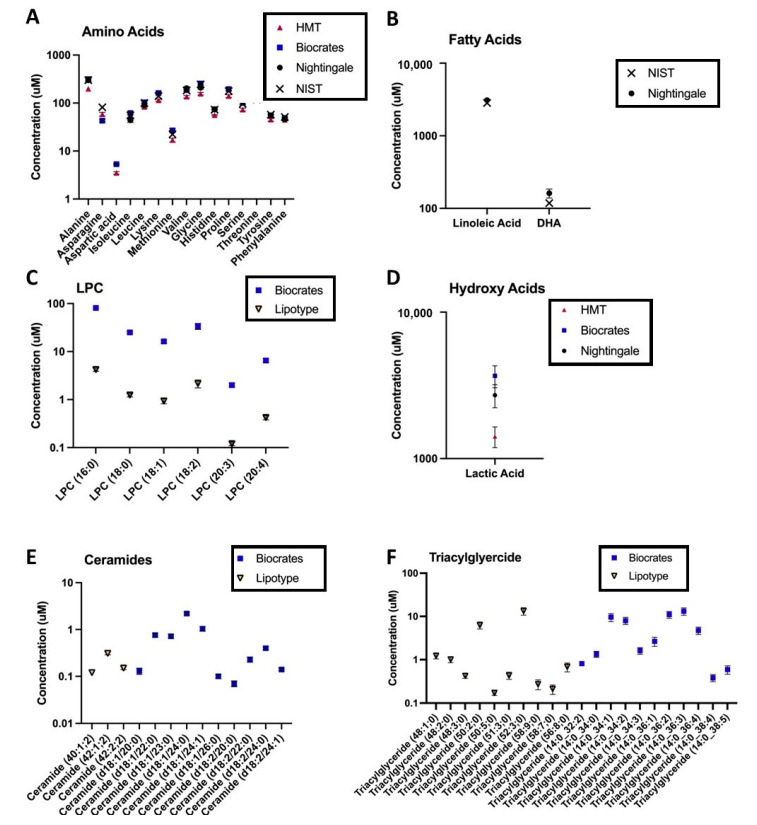
Platform-specific, log-transformed, average metabolite levels in control samples for vendors reporting absolute units; each point represents mean ± SEM for 11 control samples in total: 9 control samples from 6 individuals (with 3 technical replicates), and 2 NIST pooled reference plasma samples. Each panel depicts the range of covered metabolites, across all assays, for an exemplar metabolite class: (**A**) amino acids, (**B**) fatty acids, (**C**) lysophosphatidylcholines (LPC), (**D**) hydroxy acids, (**E**) ceramides, and (**F**) triglycerides. Depicted data are from the second sample shipment. NIST = concentrations reported in the National Institute of Standards and Technology (NIST) SRM 1950 Certificate of Analysis (COA, revised June 2020).

## Text Correction

There was an error in the original publication. NIST standards were used for part of the study to compare the accuracy of the different measurements. In the measurement of fatty acids and cholesterol, the NIST standards reflect the total concentration of fatty acids and cholesterol. The vendors in the published manuscript were reporting free fatty acids and free cholesterol, and therefore the comparison represented in Table 3 is not valid. This discrepancy was brought to our attention after the paper was published. The fatty acid and cholesterol values and percent accuracy for Biocrates and Lipotype were removed, and mention of these measurements was removed from the results section.

Updated links and data location information have been added to the appropriate sections.

A correction has been made to

1. Section 2.4, First Paragraph:

The accuracy of metabolite measurements, in comparison to NIST SRM 1950 pooled reference plasma reference values in the NIST certificate of analysis (COA), is provided in Table 3. Assessments of accuracy were constrained by (i) the fraction of classes represented in the NIST COA, (ii) vendor-specific coverage of metabolites, and (iii) the use of relative units which excluded Metabolon. Accuracy was evaluated for a set of amino acids listed in the NIST COA, which showed roughly similar high or moderate accuracy across plat-forms. Normalization methods informed by platform-specific normative levels could inform efforts to compare or merge datasets across metabolomics approaches. The majority of metabolites across all platforms were detected with excellent linearity across the dilution curve (i.e., coefficient of determination values near 1, suggesting that abundance is not a core obstacle in current metabolomics technologies; depicted in Supplementary Figure S1).

2. Supplementary Materials:

The following are available online at https://www.mdpi.com/article/10.3390/metabo11090609/s1, Figure S1: Linearity across the dilution curve of NIST pooled reference plasma (SRM 1950), Table S1: Metabolites and lipids implicated in posttraumatic stress disorder (PTSD) in previously published metabolomics studies, Table S2: List of samples sent to each metabolomics vendor in two identical shipments, Table S3: Metabolomics bake-off sample information. 

3. Data Availability Statement: 

Data and the Metabolomics Platform Exploration Tool will be made available in the BRAIN Commons, a cloud-based platform for computational discovery designed for the brain health community at https://www.braincommons.org/publications/doi-10-3390-metabo-11090609/ accessed on 28 September 2022.

The authors state that the scientific conclusions are unaffected. This correction was approved by the Academic Editor. The original publication has also been updated.
