# Supplementary material for: Cross-Platform Evaluation of Commercially Targeted and Untargeted Metabolomics Approaches to Optimize the Investigation of Psychiatric Disease
Source: Metabolites. 2021 Sep 8;11(9):609. doi: 10.3390/metabo11090609 (PMC8466258; doi:10.3390/metabo11090609)
Supplement: Supplementary file 1 [file metabolites-11-00609-s001.zip › metabolites-1346256-supplementary.pdf]

Supplementary Materials

Supplementary Figures:

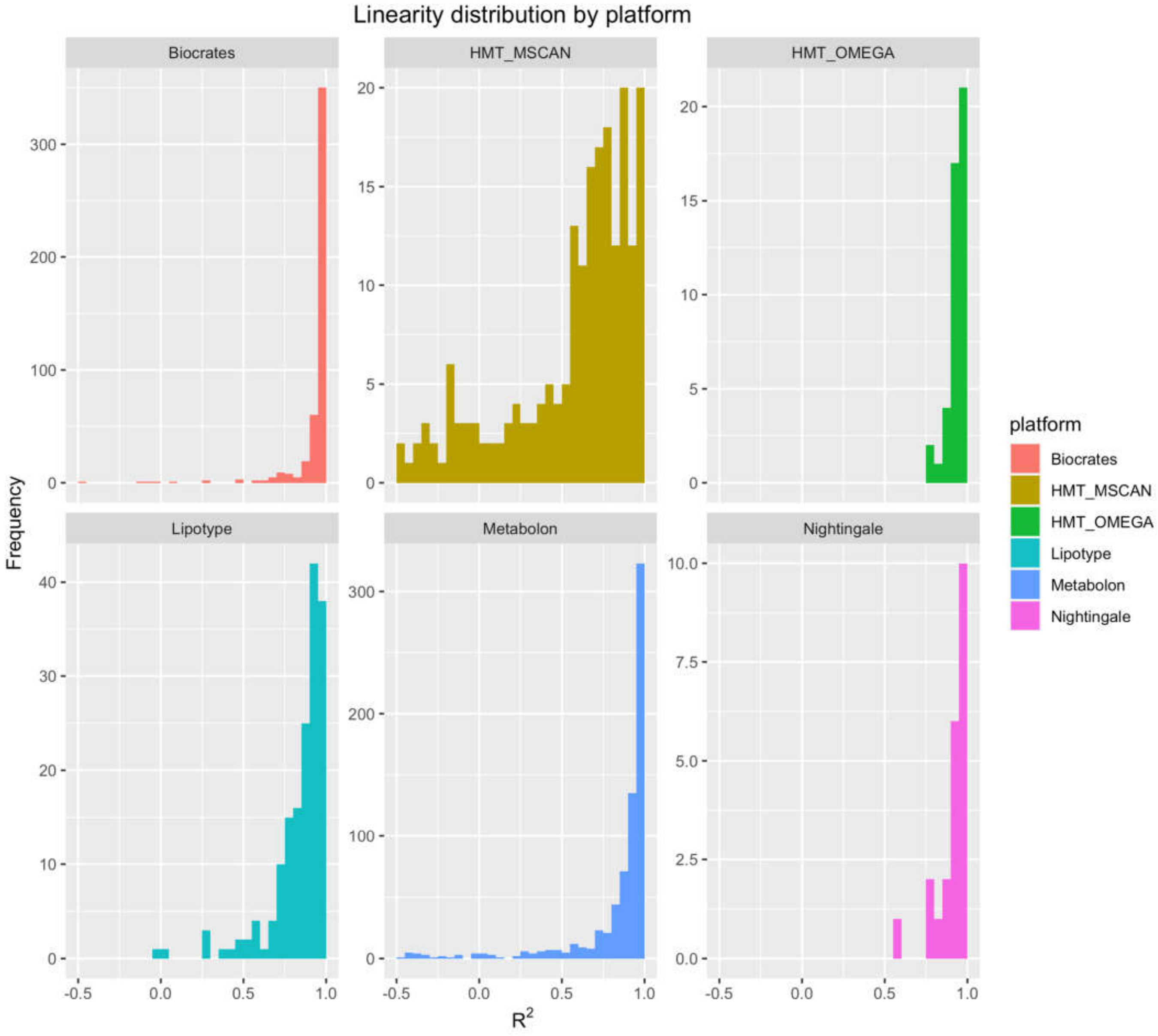

Supplementary Figure 1: Linearity across the dilution curve of NIST pooled reference plasma (SRM 1950). Values depicted are the coefficient of determination (R^2) for each metabolite reported by each vendor using all points in the dilution curve. $R^2 = 1$ for a dilution curve that is perfectly linear and extrapolates through the origin. $R^2 = 0$ indicates no relationship between dilution and reported concentration. The majority of metabolites were detected with excellent linearity, as illustrated by the density of R^2 values near 1.

**Note: Unknown metabolites were omitted, and a metabolite was excluded if it had missing values in the diluted samples for that platform.*

Supplementary Tables:

S Table 1: List of samples sent to each metabolomics vendor in two identical shipments

Sample number (inclusive of both shipments)	Sample clinical group and ID (PTSD, HC, NIST)	Other information (Sex and dilution)	Technical replicate ID	Shipment # containing sample
**1**	PTSD Sample 1	Female	A	1
**2**			B	1
**3**			C	2
**4**			D	2
**5**	PTSD Sample 2	Female	A	1
**6**			B	1
**7**			C	2
**8**			D	2
**9**	PTSD Sample 3	Female	A	1
**10**			B	2
**11**	PTSD Sample 4	Male	A	1
**12**			B	1
**13**			C	2
**14**			D	2
**15**	PTSD Sample 5	Male	A	1
**16**			B	2
**17**	PTSD Sample 6	Male	A	1
**18**			B	2
**19**	Healthy Control Sample 7	Female	A	1
**20**			B	1
**21**			C	2
**22**			D	2
**23**	Healthy Control Sample 8	Female	A	1
**24**			B	1
**25**			C	2
**26**			D	2
**27**	Healthy Control Sample 9	Female	A	1
**28**			B	2
**29**	Healthy Control Sample 10	Male	A	1
**30**			B	1
**31**			C	2
**32**			D	2
**33**	Healthy Control Sample 11	Male	A	1
**34**			B	2
**35**	Healthy Control Sample 12	Male	A	1
**36**			B	2
**37**	NIST Plasma Pool	100% (undiluted)	A	1
**38**			B	1
**39**			C	2
**40**			D	2
**41**	NIST Plasma Pool	80% (saline diluted)	A	1
**42**			B	2
**43**	NIST Plasma Pool	60% (saline diluted)	A	1
**44**			B	2
**45**	NIST Plasma Pool	40% (saline diluted)	A	1
**46**			B	2

Supplementary Table 2: Metabolomics Bake-off Sample Information

**Sample ID**	**Sex**	**Age**	**Month/year of storage***
PTSD Sample 1	Female	25	10/18
PTSD Sample 2	Female	26	10/18
PTSD Sample 3	Female	27	10/18
PTSD Sample 4	Male	39	4/18
PTSD Sample 5	Male	27	7/18
PTSD Sample 6	Male	34	10/18
Healthy Control Sample 7	Female	35	12/19
Healthy Control Sample 8	Female	30	12/19
Healthy Control Sample 9	Female	29	11/19
Healthy Control Sample 10	Male	25	11/19
Healthy Control Sample 11	Male	31	11/19
Healthy Control Sample 12	Male	29	11/19

*Clinical and control samples could not be matched on storage duration as it was necessary to prioritize either collection method or storage duration, and collection method (Vacutainer) was deemed a higher priority.

Supplementary Table 3: Metabolites and lipids implicated in posttraumatic stress disorder (PTSD) in previously published metabolomics studies

Metabolites	Direction in PTSD*	Citation+
**Hormones**		
(1) Cortisol	Increase	(Mellon et al., 2019)
(2) Epinephrine	Increase	(Somvanshi et al., 2019)
**Nucleotides**		
(3) Hypoxanthine	Increase	(Somvanshi et al., 2019)
	Increase	(Mellon et al., 2019)
**Nucleosides**		
(4) Guanosine	Decrease	(Karabatsiakis et al., 2015)
(5) Inosine	Decrease	(Karabatsiakis et al., 2015)
**Bile acids and derivatives**		
(6) 3α-hydroxy-5β-cholan-24-oic acid	Decrease	(Karabatsiakis et al., 2015)
(7) 7α,12α-dihydroxy-3-oxocholest-4-en-26-oic acid	Increase	(Karabatsiakis et al., 2015)
(8) Glycocholic Acid	Decrease	(Karabatsiakis et al., 2015)
**Glycolysis**		
(9) Glucose	Increase	(Somvanshi et al., 2019)
(10) Lactate	Increase	(Somvanshi et al., 2019)
	Increase	(Dean et al., 2019)
	Increase	(Mellon et al., 2019)
(11) Pyruvate	Increase	(Somvanshi et al., 2019)
	Increase	(Mellon et al., 2019)
(12) Citrate	Decrease	(Somvanshi et al., 2019)
	Increase	(Dean et al., 2019)
	Decrease	(Mellon et al., 2019)
**Amino acids**		
(13) Alanine	Increase	(Somvanshi et al., 2019)
(14) Glutamine	Decrease	(Somvanshi et al., 2019)
	Decrease	(Mellon et al., 2019)
(15) Tyrosine	Increase	(Somvanshi et al., 2019)
(16) Isoleucine	Increase	(Somvanshi et al., 2019)
(17) Trans-urocanate	Decrease	(Mellon et al., 2019)
(18) Phenyllactate (PLA)	Increase	(Mellon et al., 2019)
(19) 3-hydroxyisobutyrate	Decrease	(Mellon et al., 2019)
(20) Proline	Increase	(Konjevod et al., 2020)#
(21) 4-hydroxyproline	Increase	(Konjevod et al., 2020)#
(22) Aminomalonic acid	Increase	(Konjevod et al., 2020)#
**Organooxygen compounds**		
(23) Kynurenine	Decrease	(Smith et al., 2020)
**Hydroxy acids**		

(24) Malic acid	Increase	(Konjevod et al., 2020)#
**Urea cycle**		
(25) Arginine	Decrease	(Somvanshi et al., 2019)
	Mixed across test sets	(Dean et al., 2019)
	Decrease	(Mellon et al., 2019)
(26) Ornithine	Increase	(Somvanshi et al., 2019)
**Carnitines**		
(27) Carnitine	Decrease	(Dean et al., 2019)
(28) Decanoylcarnitine	Increase	(Somvanshi et al., 2019)
	Increase	(Mellon et al., 2019)
(29) Octanoylcarnitine	Increase	(Somvanshi et al., 2019)
	Increase	(Mellon et al., 2019)
(30) Hexanoylcarnitine	Increase	(Mellon et al., 2019)
(31) Linoelaidyl carnitine	Decrease	(Konjevod et al., 2020)^
(32) Stearoylcarnitine	Decrease	(Konjevod et al., 2020)^
**Anti-Oxidants**		
(33) Pantothenic Acid	Decrease	(Karabatsiakis et al., 2015)
**Monosaccharides**		
(34) N-Acetylglucosamine-6-phosphate	Decrease	(Karabatsiakis et al., 2015)
**Carbohydrates**		
(35) Fructose	Increase	(Konjevod et al., 2020)#
**Quaternary ammonium salts**		
(36) Choline	Increase	(Konjevod et al., 2020)#
**Dicarboxylic acids**		
(37) 3,7-Dimethyl-2E,6E-decadien-1,10-dioic acid	Increase	(Konjevod et al., 2020)#
**Lipids**		
**Long chain fatty acids**		
(38) 10-Nonadecenoate 19:1 (ω-9)	Decrease	(Somvanshi et al., 2019)
(39) Nonadecanoate 19:0	Increase	(Mellon et al., 2019)
(40) Arachidonate 20:4 (ω-6)	Decrease	(Somvanshi et al., 2019)
(41) Hydroxy stearic acid	Increase	(Konjevod et al., 2020)^
**Essential fatty acids**		
(42) Dihomolinoleate 20:2 (ω-6)	Decrease	(Mellon et al., 2019)
(43) Dihomolinolenate 20:3 (ω-3 or ω-6)	Decrease	(Mellon et al., 2019)
(44) Docosahexaenoate (DHA) 22:6 (ω-3)	Decrease	(Mellon et al., 2019)
(45) Eicosenoate 20:1	Decrease	(Mellon et al., 2019)
(46) Palmitoylethanolamide	Decrease	(Karabatsiakis et al., 2015)
(47) Linolenate 18:3 (ω-3 or ω-6)	Decrease	(Mellon et al., 2019)
(48) Palmitic amide	Decrease	(Karabatsiakis et al., 2015)
**Fatty acid esters**		
(49) Arachidonic acid methyl ester	Increase	(Konjevod et al., 2020)#

**Glycerophospholipids**		
(50) PE(0:0/16:0)	Increase	(Konjevod et al., 2020)[#]
(51) PE(17:1(9Z)18:0)	Increase	(Karabatsiakis et al., 2015)
(52) PE(P-18:1(11Z)/15:0)	Increase	(Karabatsiakis et al., 2015)
(53) PE(18:1/0:0)	Increase	(Konjevod et al., 2020)^[#]
(54) PE-Nme(O-14:0/O-14:0)	Increase	(Karabatsiakis et al., 2015)
(55) PE-NMe2(O-14:0/O-14:0)	Increase	(Karabatsiakis et al., 2015)
**Glycerophosphocholines**		
(56) PC(16:0/0:0)	Increase	(Konjevod et al., 2020)[#]
(57) PC(16:1/0:0)	Increase	(Konjevod et al., 2020)^
(58) PC(16:2/0:0)	Increase	(Konjevod et al., 2020)[#]
(59) PC(17:0/0:0)	Increase	(Konjevod et al., 2020)[#]
(60) PC(18:0/0:0)	Decrease	(Konjevod et al., 2020)[#]
(61) PC(18:1/0:0)	Increase	(Konjevod et al., 2020)^[#]
(62) PC(19:0/0:0)	Increase	(Konjevod et al., 2020)[#]
(63) PC(20:4/0:0)	Increase	(Konjevod et al., 2020)[#]
(64) PC(22:4/0:0)	Increase	(Konjevod et al., 2020)[#]
(65) PC(O-16:1/0:0)	Increase	(Konjevod et al., 2020)[#]
(66) PC(P-18:0/2:0)	Increase	(Konjevod et al., 2020)[#]
(67) PC(P-18:1/0:0)	Increase	(Konjevod et al., 2020)[#]
**Glycerophosphoinositols**		
(68) PI(18:1/0:0)	Increase	(Konjevod et al., 2020)[#]
**Triglycerides**		
(69) Triglycerides	Increase	(Somvanshi et al., 2019)
(70) TG(46:1)		(Huguenard et al., 2020)
(71) TG(54:6)		(Huguenard et al., 2020)
**Phosphatidylserines**		
(72) Phosphatidylserine (36:2)		(Huguenard et al., 2020)
**Oxidative Stress & Inflammation**		
(73) Gammaglutamyltyrosine	Increase	(Dean et al., 2019)
(74) 5-Oxoproline	Increase	(Mellon et al., 2019)
(75) Sphyingosine 1 phosphate	Increase	(Mellon et al., 2019)
(76) Sphingosine 1-phosphate	Decrease	(Konjevod et al., 2020)^
TNF-a / IL6 / CRP	Increase	(Somvanshi et al., 2019)

* Versus controls if available

[+] The publications Somvanshi et al., 2019 and Dean et al., 2019 specify that they use the same data, and Mellon et al., 2019 appear to use the same data with an additional ~30 subjects compared with Somvanshi et al., 2019 and Dean et al., 2019 such that replication in these studies may not represent independent replication

^ indicates found in exploratory cohort

[#] indicates found in validation cohort
